# A High-Resolution View of Adaptive Event Dynamics in a Plasmid

**DOI:** 10.1093/gbe/evz197

**Published:** 2019-09-18

**Authors:** Han Mei, Barbara Arbeithuber, Marzia A Cremona, Michael DeGiorgio, Anton Nekrutenko

**Affiliations:** 1 Department of Biochemistry and Molecular Biology, The Pennsylvania State University; 2 Department of Biology, The Pennsylvania State University; 3 Department of Statistics, The Pennsylvania State University; 4 Department of Operations and Decision Systems, Université Laval; 5 Institute for CyberScience, The Pennsylvania State University

**Keywords:** adaptation, experimental evolution, duplex sequencing, plasmids

## Abstract

Coadaptation between bacterial hosts and plasmids frequently results in adaptive changes restricted exclusively to host genome leaving plasmids unchanged. To better understand this remarkable stability, we transformed naïve *Escherichia coli* cells with a plasmid carrying an antibiotic-resistance gene and forced them to adapt in a turbidostat environment. We then drew population samples at regular intervals and subjected them to duplex sequencing—a technique specifically designed for identification of low-frequency mutations. Variants at ten sites implicated in plasmid copy number control emerged almost immediately, tracked consistently across the experiment’s time points, and faded below detectable frequencies toward the end. This variation crash coincided with the emergence of mutations on the host chromosome. Mathematical modeling of trajectories for adaptive changes affecting plasmid copy number showed that such mutations cannot readily fix or even reach appreciable frequencies. We conclude that there is a strong selection against alterations of copy number even if it can provide a degree of growth advantage. This incentive is likely rooted in the complex interplay between mutated and wild-type plasmids constrained within a single cell and underscores the importance of understanding of intracellular plasmid variability.

## Introduction

Bacterial plasmids are self-replicating genetic elements that are fundamentally important to the evolution and adaptability of their hosts. Plasmids exhibit a wide variety of sizes, replication strategies, and transmission modes (reviewed in Tolmasky and Alonso 2015). Some of the best known are small, multicopy derivatives of *Escherichiacoli* ColE1 plasmid exemplified by widely used cloning vectors such as pBR322 (Bolivar et al. 1977) or pUC family (Yanisch-Perron et al. 1985) and their numerous descendants. These plasmids exist in multiple copies per cell and their segregation into daughter cells upon division is random—it is not controlled by an active partitioning system characteristic of large, low-copy number plasmids. They cannot be horizontally transferred by conjugation or mobilized via a *trans*-acting conjugative element. In addition to being important for biotechnology, small multicopy plasmids are highly medically relevant. The majority of plasmids in clinical isolates such as the epidemic clone ST131 are small ColE1-like replicons (Stoesser et al. 2016). Moreover, in *E. coli* small, stochastically segregating nonconjugative and nonmobilizable plasmids account for approximately half of all known plasmids (De Toro et al. 2015).

Studies of evolutionary dynamics playing out between small multicopy plasmids and their bacterial hosts underscored their remarkable stability. Classical works of Helling et al. (1981) and Lenski (Lenski and Bouma 1987; Bouma and Lenski 1988; Lenski et al. 1994) showed that the coadaptation of a naïve host and a newly introduced plasmid occurs entirely at the expense of the host genome with plasmid remaining unchanged. Subsequent use of high-throughput sequencing for study of coadaptation between similarly small, high-copy number, nonconjugative plasmids and a host genome did not find alterations within the plasmid genome (San Millan et al. 2014; Ilhan et al. 2019). A similar observation, no changes in the plasmid genome, was made in cases of much larger replicons such as RP4 (Loftie-Eaton et al. 2017) or pQBR103 (Harrison et al. 2015) that were introduced into hosts where these plasmids were known to be unstable and are easily lost. A common feature of these otherwise distinct studies is that they all employed plasmids that are rapidly lost from host cells in the absence of selection for functions encoded on them. This loss is due to uneven partitioning of plasmids between daughter cells after division, which in turn is caused by either complete lack of partitioning mechanism or its deficiency in a given plasmid/host combination. As a result, plasmid-free daughter cells produced through segregational loss free themselves from metabolic burden imposed by plasmid carriage and quickly outcompete cells containing the plasmid.

The apparent lack of changes in small multicopy plasmids in coadaptation experiments is likely due to their segregation dynamics. To illustrate this point, envision a bacterial cell with a 10^6^ nucleotide chromosome carrying 10 copies of a 1,000 nucleotide plasmid. Assuming a mutation rate of $10^{-9}$ per site per generation (as in *E. coli*; Drake 1991; Wielgoss et al. 2011; Lee et al. 2012; Jee et al. 2016), the rate per genome and per plasmid will be $10^{-3}$ ($10^{-9}\times 10^{6}$) and $10^{-5}$ ($10^{-9}\times 1000\times 10$), respectively. In other words, in a given generation 1 out 1,000 cells is expected to incur a chromosomal mutation, while for the plasmid this number is 1 out of 100,000 molecules. Because there is no horizontal transfer, each plasmid is confined to its own lineage of dividing cells. In such a lineage, after cell division, the chromosomal mutation will be partitioned between daughter cells, while the fate of plasmid mutation will depend on its replication efficiency and random partitioning at doubling. If a chromosomal mutation has a positive effect on fitness, then it will sweep to fixation. Numerically ($10^{-3}$ vs. $10^{-5}$), a chromosomal mutation is 1) more probable and 2) will likely occur at the wild-type plasmid background leaving the latter to appear unchanged after the sweep is complete. In addition, clonal interference across multiple plasmids within an individual cell may further diminish the number of observable plasmid mutations (Bedhomme et al. 2017). These factors may potentially explain perceived constancy of plasmids in experiments by Lenski et al. (1994) and San Millan et al. (2014). One way to change this balance is to increase the copy number of the plasmid, which will in turn increase the supply of plasmid mutations. Ilhan et al. (2019) examined this possibility. These authors introduced low- and high-copy number plasmids into two *E. coli* strains (wild-type and hypermutator) and evolved them for 800 generations in a continuous culture with ampicillin selection at two temperatures. Although multiple mutations were found within the bacterial chromosome, no mutations were found within plasmids regardless of their copy number. Ilhan et al. (2019) used simulations to show that if a plasmid mutation confers a selective advantage, then it eventually fixes, but the time to fixation is longer for high-copy number plasmids.

One potential limitation of the previous studies is low resolution of methods used to detect sequence variants. Lenski et al. (1994) did not employ sequencing and instead used ancestral plasmid on evolved host background to demonstrate that the host genome alone is responsible for coadaptation. Studies employing next-generation sequencing (San Millan et al. 2014; Harrison et al. 2015; Loftie-Eaton et al. 2017; Ilhan et al. 2019) used standard approaches for identification of sequence variants based on aligning the reads against reference genomes. However, these techniques only allow the observation of evolutionary trajectories of mutations that have risen above the frequency of ∼1%—a threshold at which the noise of Illumina sequencing (the most accurate of the currently available sequencing technologies) obscures the signal (Rebolledo-Jaramillo et al. 2014; Salk et al. 2018). Such a threshold is too high to obtain reliable insight into the underlying genetic variability within a large population. For example, San Millan et al. (2014) used 2 ml of the overnight bacterial culture for detecting changes responsible for coadaptation between *Pseudomonasaeruginosa* PA01 and plasmid pNUK73. This volume conservatively contains $∼10^{8}$ cells. Considering the mean estimated pNUK73 copy number of 11 (San Millan et al. 2014), this amount of cells contains $∼10^{9}$ plasmid molecules. Thus, a variant detection threshold of ∼1% will miss all variants present in fewer than $∼10^{7}$ plasmids! However, if we recall the simplistic numerical argument made above—a mutation rate of 10^5^ per plasmid per generation for a plasmid with copy number of 10—a population of 10^8^ cells containing 10^9^ plasmids will gain 10^4^ mutations in one generation. The vast majority of these mutations will be deleterious yet still there must be an interesting dynamics that is simply below the resolution of the current methods. We know this from studies that employed molecular barcodes within an experimental evolution setting (Levy et al. 2015; Venkataram et al. 2016; Blundell et al. 2019). These authors have shown that at the beginning of an experiment, a large pool of lineages carrying beneficial mutations is present within the frequency band between $10^{-8}$ and $10^{-4}$, and a vast majority of adaptive lineages have frequencies under 0.01% (well below what is detectable with conventional Illumina sequencing). These data provided us with invaluable insight into early adaptive events. However, they do not convey the nature of genetic variants, as the barcode counts are only proxies for the number of cells carrying a particular, yet unknown, beneficial mutation. Venkataram et al. (2016) recognized this limitation and used barcoding to select several hundred lineages carrying beneficial mutations for subsequent whole-genome sequencing. Still, this is only the tip of the iceberg, with the majority of variants remaining below the surface.

A number of techniques have been recently developed to allow sequencing-based detection of very rare genetic changes that may be suitable to directly observe early adaptive events. Of these, duplex sequencing is the most sensitive, with a theoretical resolution threshold of $<10^{-7}$ (Schmitt et al. 2012; Salk et al. 2018). It is based on using unique sequence tags to label individual molecules of the input DNA prior to preparation of Illumina sequencing libraries. During the amplification steps of library preparation, each of these molecules gives rise to multiple descendants. After sequencing, the descendants of each original DNA fragment are identified and grouped together using tags—that is, one simply sorts tags in sequencing reads lexicographically, and all reads containing the same tag are bundled into families. These families (usually with at least three members) form single-strand consensus sequences (SSCS) for the forward or the reverse strand, respectively. Complementary SSCS are then grouped to produce duplex consensus sequences (DCS). A true sequence variant is present in all reads within a family forming a duplex. In contrast, sequencing and amplification errors will manifest themselves as “polymorphisms” within a family, and so they can be identified and reliably removed.

Our current knowledge of the plasmid/host dynamics implies several interesting phenomena. First, segregational drift reduces fixation time for beneficial mutations but at the same time likely provides “cushioning” against deleterious mutations not unlike the mitochondrial bottleneck in eukaryotic cells (Bergstrom and Pritchard 1998). These effects are responsible for the apparent stability of plasmids. Second, studies using molecular barcodes suggest that underlying situation is likely more complex at the level of individual replicons where numerous low-frequency coexisting variants may compete with each other through clonal interference (Gerrish and Lenski 1998; Desai and Fisher 2007; Miller et al. 2011). Our goal was to reconcile these predictions by tracing plasmid mutations as they emerge during a coevolution experiment using duplex sequencing.

## Materials and Methods

### Strains and Plasmids

*Escherichia* *coli* strain DH5*α* was obtained from Invitrogen (cat. 18265017). The cells were transformed with plasmid pBR322 (NEB cat. N3033S) according to the manufacturer’s protocol. pBR322 carries the replication origin, *tetC*, encoding a tetracycline efflux pump, *bla*, conferring resistance to ampicillin, and *rop/rom*, controlling replication (Eguchi and Tomizawa 1990). All experiments were performed in LB broth (EMD cat. 110285), including those in turbidostat and agar plates, that was supplemented with 30 µg/ml tetracycline (Sigma–Aldrich cat. T3258) and 0.05% antifoam B (Sigma–Aldrich cat. A5757). Transformants were spread over a LB agar plate. A series of single colonies were picked, grown in LB, and then stored in 20% glycerol stock in a −80 °C freezer. One glycerol stock was used as the founder to inoculate all turbidostat runs.

### Turbidostat Set-up and Experimental Evolution

The turbidostat set-up was described by Takahashi et al. (2015). It consists of three connected parts (supplementary fig. S8, Supplementary Material online): a carboy containing LB medium, a bacteria growing chamber that is revolved magnetically, and a waste tank. The carboy and the chamber were pressurized by an aquarium air pump. The volume in the chamber was kept constant at 13 ml. OD_600_ was measured in 30-s intervals and LB was added as necessary to maintain OD_600_ constant at 0.8.

About 50 µl of the glycerol stock were inoculated into 13 ml of LB medium and grown for 14 h at 250 rpm, 37 °C. A 4-ml cell culture was inoculated into turbidostat. A 1-ml culture was frozen as glycerol stock in −80 °C as the initial sample. The remaining culture was labeled as time point 0 and collected by centrifugation at 4,000 *g* for 5 min. The pellet was then transferred to −20 °C. Turbidostat runs were carried out in an incubator to maintain 37 °C. A 8-ml culture was taken from the turbidostat at regular 12-h intervals, which constitute time points for the subsequent analysis. The turbidostat was immediately refilled by fresh broth to the 13 ml mark. When the turbidostat run was completed, the terminal sample was also collected and archived as glycerol stock in −80 °C.

### Population Fitness Estimation

OD_600_ values were constantly monitored, and growth curves were inferred from them. We found that exponential growth between OD_600_ values of 0.6–0.8 showed the steepest slope on the growth curve. Therefore, we used it as a proxy for overall population fitness. The maximum growth rate was defined as follows:

$$\frac{\ln(\frac{0.8}{0.6})}{t}$$

where *t* is the duration in minutes in which OD_600_ values increased from 0.6 to 0.8 (Zwietering et al. 1990). The generation time was calculated as $\frac{\ln(2)}{\mathrm{growth}\;\mathrm{rate}}$ (Lee et al. 2013). We found that the generation time varied over the course of the two long-term replicates. Detailed information regarding growth rate and generation time are displayed in supplementary table S2, Supplementary Material online. For simplicity, the generation time was assumed as 60 min when we modeled the transmission of plasmid heteroplasmy.

### Fitness remeasurement of samples representing the initial and terminal time points

About 50 µl of the glycerol stock from each of the initial and terminal samples from the two long-term replicates R6 and R7 were inoculated into turbidostat in triplicate. OD_600_ was monitored and growth curves were inferred. Exponential growth between OD_600_ values of 0.6–0.8 was used to estimate fitness.

### pBR322 Curing

About 50 µl of bacterial glycerol stock were grown in 3 ml LB at 220 rpm for 24 h at 37 °C. Six additional 1,000-fold serial transfers were performed in 3 ml LB with a 24-h growth period. Bacterial culture from the fourth of the seven transfers (Chiang and Bremer 1988) was streaked on a Bochner/Maloy plate (Maloy and Nunn 1981) to select for bacterial colonies losing pBR322. Three colonies from a Bochner/Maloy plate were picked, grown in LB, streaked on three LB plates, respectively, to repurify single colonies. One colony picked from each of the three LB plates was considered pBR322-cured if no growth was observed in LB supplemented with tetracycline and pBR322 was unable to be amplified by PCR. *treB* and *recA* mutations were verified in pBR322-cured strains by Sanger sequencing. Fitness of pBR322-cured clones in LB were indicated by the exponential growth between OD_600_ values of 0.6–0.8 (table 1) and calculated as mentioned above. This pBR322 curing protocol is schematically shown in supplementary figure S5, Supplementary Material online.

**Table 1:** Growth Rates with and without the Plasmid in LB Medium

Host	Plasmid	Replicates		
		1	2	3
Original DH5*α*	−	0.0079	0.0080	0.0082
R6-S0	+	0.0077	0.0077	0.0074
R7-S0	+	0.0071	0.0071	0.0072
R6-S27	+	0.0170	0.0166	0.0165
R7-S27	+	0.0092	0.0085	0.0085
R6-S27-cured	−	0.0177	0.0189	0.0179
R7-S27-cured	−	0.0187	0.0181	0.0171

### Duplex Sequencing and Calling Plasmid Variants

Plasmid at selected time points was extracted by mini-prep (Qiagen cat. 27104). Duplex-sequencing library preparation and variant calling were performed as previously described (Rebolledo-Jaramillo et al. 2014; Stoler et al. 2016). Briefly, 100 ng of plasmid was sheared to 550 bp on a M220 platform (Covaris) according to the manufacturer’s instructions at the Pennsylvania State University Genomics Core Facility. The sheared DNA was subjected to end-repair (NEB cat. E6050S), size selection (Beckman Coulter cat. A63881), 3′-end dA-tailing (NEB cat. M0212L), duplex adaptor ligation (NEB cat. M0202T), and PCR amplification (Kapa Biosystems cat. kk2612). PCR amplicons were quantified by qPCR (Kapa Biosystems cat. kk4873) and sequenced on an Illumina MiSeq platform using 301-nt paired-end reads. DCS were generated from raw reads and mapped to the reference to call variants. The variant calling workflow is available on Galaxy (Goecks et al. 2010) at https://usegalaxy.org/u/hanmei/w/du-var-calling, last accessed September 19, 2019.

### Whole-Genome Sequencing and Calling Chromosomal Variants

Genomic DNA was extracted (Qiagen cat. 51304) from samples representing the initial and terminal time points of the two long-term replicates. Whole-genome sequencing was performed as described in our previous article (Lariviere et al. 2018). Briefly, sequencing libraries were prepared as described for duplex libraries, except that 2 µg of DNA was used as the starting material for shearing and adaptors from the Illumina TruSeq Kit were used. Without PCR amplification, ligated libraries were directly subjected to MiSeq sequencing. Variants were called against the DH5α reference genome (GenBank accession number CP017100) using the haploid variant calling workflow on Galaxy at https://usegalaxy.org/u/hanmei/w/haploid-var-calling, last accessed September 19, 2019.

### Data Modeling

#### Initialization

At generation 0, the population starts out with cells containing 0 chromosomal mutations, 20 wild-type plasmids, and 0 mutated plasmids so that $n_{0}(0,20,0)=n$ where *n* is the predetermined size of the population.

#### Division

##### 

(**Plasmid Diffusion**) At generation *t*, we have a certain number of mature cells $n_{t}(g,w,m)$ with *w* wild-type and *m* mutant plasmids, and where *g *=* *0 if the cell has no chromosomal mutation and *g *=* *1 if the cell has a chromosomal mutation. To initiate movement to generation *t *+* *1, these cells then must divide into two daughter cells, for which their *w* wild-type and *m* mutant plasmids will diffuse into. Upon division, one daughter cell will have j∈{0,1,…,w} wild-type plasmids and k∈{0,1,…,m} mutant plasmids, whereas the other daughter cell will have *w*−*j* wild-type plasmids and *m*−*k* mutant plasmids. The probability that one daughter cell has *j *+* k* total plasmids is binomial-distributed with *w *+* m* trials, *j *+* k* successes, and success probability 1/2:

$$B(w+m,j+k,1/2)=\left(\begin{array}{c} w+m\\ j+k \end{array}\right)\frac{1}{2^{w+m}},$$

and this scenario corresponds to a circular cell with independent plasmids uniformly distributed across the cell that is cut in half during division. Conditional on these *j *+* k* plasmids, the probability that the daughter cell will have *j* wild-type plasmids out of a total of *j *+* k* plasmids is hypergeometrically distributed with probability:

$$\text{HG}(j,k;w,m)=\frac{\left(\begin{array}{c} w\\ j \end{array}\right)\left(\begin{array}{c} m\\ k \end{array}\right)}{\left(\begin{array}{c} w+m\\ j+k \end{array}\right)}.$$

That is, for *g *=* *0 and 1, $w=0,1,\ldots ,\kappa_{T}-1$ and $m=0,1,\ldots ,\kappa_{T}-1-w$, after division at generation *t *+* *1 we have:

$$n_{t+1}^{\text{div}}(g,w,m)=\sum_{a=0}^{\kappa_{T}-1}\sum_{b=0}^{\kappa_{T}-1-a}n_{t}(g,a,b)\sum_{j=0}^{a}\sum_{k=0}^{b}B(a+b,j+k,1/2)\text{HG}(j,k;a,b)(1_{\left\{j=w,k=m\right\}}+1_{\left\{a-j=w,b-k=m\right\}}).$$

#### Replication

(**Chromosomal Mutation**) Chromosomal mutations are introduced during the cell division process where chromosomes are replicated. We will let chromosomal mutations occur on nonchromosomal-mutated backgrounds *g* = 0 at rate *γ*. That is, for *g* = 0 and 1, $w=0,1,\ldots ,\kappa_{T}-1$ and $m=0,1,\ldots ,\kappa_{T}-1-w$ at generation *t* + 1, after mutation we will now have:

$$\begin{array}{c} n_{t+1}^{\text{mut chrom}}(1,w,m)=\gamma n_{t+1}^{\text{div}}(0,w,m)\\ n_{t+1}^{\text{mut chrom}}(0,w,m)=(1-\gamma )n_{t+1}^{\text{div}}(0,w,m). \end{array}$$

(**Plasmid Replication**) After division and chromosomal mutation, we now have a certain number of cells $n_{t+1}^{\text{mut chrom}}(g,w,m)$ in the next generation with *w* wild-type and *m* mutant plasmids on a chromosomal mutation background if *g* = 1 and not on a chromosomal mutation background if *g* = 0. These cells then undergo plasmid replication, which involves an increase in the number of wild-type and mutant plasmids at rates *r* and *R*, respectively, as well as mutation from wild-type to mutant plasmids with probability *μ* per wild-type plasmid. That is, for *g* = 0 and 1, $w=0,1,\ldots ,\kappa_{T}-1$ and $m=0,1,\ldots ,\kappa_{T}-1-w$, after plasmid replication at generation *t* + 1 we have:

$$n_{t+1}^{\text{rep plasmid}}(g,w,m)=\sum_{a=0}^{\kappa_{T}-1}\sum_{b=0}^{\kappa_{T}-1-a}n_{t+1}^{\text{mut chrom}}(g,a,b)1_{\left\{\left\lfloor \mathrm{ar}\right\rfloor =w,\left\lfloor \mathrm{bR}\right\rfloor =m\right\}}$$

(**Plasmid Mutation**) During plasmid replication, the number of wild-type plasmids in a cell generally increases. The newly synthesized plasmids can undergo plasmid mutation with a rate of *μ*. For each combination of *w* wild-type and *m* mutated plasmid cells, let the *w_new_* newly synthesized wild-type plasmids mutate to give a cell with *w* – *k* wild-type and *m* + *k* mutant plasmids with binomial probability:

$$B(w_{\mathrm{new}},k,\mu )=\left(\begin{array}{c} w_{\mathrm{new}}\\ k \end{array}\right)\mu^{k}{\left(1-\mu \right)}^{w_{\mathrm{new}}-k},$$

for $k=0,1,\ldots ,w_{\mathrm{new}}$. That is for *g* = 0 and 1, $w=0,1,\ldots ,\kappa_{T}-1$ and $m=0,1,\ldots ,\kappa_{T}-1-w$, after plasmid mutation we have:

$$n_{t+1}^{\text{mut plasmid}}(g,w,m)=\sum_{a=0}^{\kappa_{T}-1}\sum_{b=0}^{\kappa_{T}-1-a}n_{t+1}^{\text{rep plasmid}}(g,a,b)\sum_{k=0}^{w_{\mathrm{new}}}B(w_{\mathrm{new}},k,\mu )1_{\left\{a-k=w,b+k=m\right\}}.$$

#### Selection

After a round of replication (chromosomal mutation, plasmid replication, and plasmid mutation), we now have a certain number of cells $n_{t+1}^{\text{mut plasmid}}(g,w,m)$ in the next generation with *w* wild-type and *m* mutant plasmids on a chromosomal mutation background if *g *=* *1 and not on a chromosomal mutation background if *g *=* *0. These cells are then selected based on a fitness function S(g,w+m)∈[0,1] (this particular form of the fitness function represents one of many potential fitness functions to describe frequency dependent selection, and it is possible that other fitness functions could also be suitable in this setting; Harmand et al. 2019). For further simplicity, we will assume the following piecewise fitness function:

$$S(g,w+m)=\{\begin{array}{cc} 1 & \text{if }g=1\text{ and }0<w+m<\kappa_{T}\\ \frac{S+(1-S)(w+m-1)}{\left(\kappa^{*}-1\right)} & \text{if }g=0\text{ and }w+m=1,2,\ldots ,\kappa^{*}\\ \frac{\left(\kappa_{T}-w-m\right)}{\left(\kappa_{T}-\kappa^{*}\right)} & \text{if }g=0\text{ and }w+m=\kappa^{*}+1,\kappa^{*}+2,\ldots ,\kappa_{T}\\ 0 & \text{if }w+m=0\text{ or }w+m>\kappa_{T} \end{array}$$

The first condition, is when the cell has a chromosomal mutation and some plasmids that are fewer in number than the threshold of *κ_T_*, and so the probability of survival is equal to the base-line 1. The second condition is when the cell has no chromosomal mutation and has some plasmids, but the number does not exceed the optimal number $\kappa^{*}$. Under this condition, increasing plasmid copy number linearly increases the survival probability from *S* to 1, up until there are $\kappa^{*}$ plasmids in which the survival probability is 1. The third condition is when the cell has no chromosomal mutation and has more plasmids than the optimal number, but the number does not exceed the threshold of *κ_T_*. Under this condition, increasing plasmid copy number linearly decreases the survival probability from 1 to 0, up until there are *κ_T_* plasmids in which the survival probability is 0. The fourth condition enforces that no cells survive if they have no plasmids or if they have too many plasmids. Therefore, for *g *=* *0 and 1, $w=0,1,\ldots ,\kappa_{T}-1$ and $m=0,1,\ldots ,\kappa_{T}-1-w$ after selection in generation *t *+* *1, we have:

$$n_{t+1}(g,w,m)=n_{t+1}^{\text{mut plasmid}}(g,w,m)S(g,w+m),$$

giving us the number of mature cells with *w* wild-type and *m* mutant plasmids in the next generation on a chromosomal mutation background if *g *=* *1 and not on a chromosomal mutation background if *g *=* *0.

#### Estimating Model Parameters

If we now denote the population frequency of cells with *w* wild-type and *m* mutant plasmids at generation *t* by,

$$p_{t}(w,m)=\frac{n_{t}(0,w,m)+n_{t}(1,w,m)}{\sum_{g=0}^{1}\sum_{a=0}^{\kappa_{T}-1}\sum_{b=0}^{\kappa_{T}-1-a}n_{t}(g,a,b)},$$

then we can obtain the proportion *β_t_* of mutant plasmids in the population at time *t* by,

$$\beta_{t}=\frac{\sum_{w=0}^{\kappa_{T}-1}\sum_{m=0}^{\kappa_{T}-1-w}mp_{t}(w,m)}{\sum_{w=0}^{\kappa_{T}-1}\sum_{m=0}^{\kappa_{T}-1-w}\left(w+m\right)p_{t}(w,m)}.$$

Given the values of the parameters, we iterate the division, replication, and selection steps over 318 generations (the duration of the long-term turbidostat run), and therefore obtained the proportion *β_t_* of mutant plasmids. We also empirically estimated the mutant frequency at the generations corresponding to the time points selected for plasmid duplex sequencing. In particular, at each of these empirically determined generations, we computed the empirical mutant frequency as the sum of frequencies of mutations in bases 3,027–3,035 and 3,118 determined in turbidostat experiments. We then defined the cost function as the sum of squared differences between *β_t_* and the empirical mutation frequencies. At each of these empirically determined generations, we computed the empirical mutant frequency as the sum of frequencies of mutations in bases 3,027–3,035 and 3,118 determined in turbidostat experiments. We denote the empirical sampling time points as $t_{i},i=1,2,\ldots ,I$. Moreover, let $x=(x_{t_{1}},x_{t_{2}},\ldots ,x_{t_{I}})$ denote the vector of sample proportions of mutant plasmids taken across the time points, where *x_t_* is the sample proportion at time *t*. Finally, we denote the vector of parameters by $\theta =(\gamma ,\mu ,r,R,S,\kappa^{*},\kappa_{T})$. We define the cost function as follows:

$$J_{\theta }(x)=\sum_{i=1}^{I}{\left(x_{t_{i}}-\beta_{t_{i}}\right)}^{2},$$

nad infer the model parameters that minimize it. This cost function was optimized over the parameters using the R function optim, setting bounds on the parameters as shown in table 2 (choice of these bound is discussed in the next paragraph). Note that optim was run with a maximum of 10^6^ iterations in order to facilitate convergence of the optimization process. Since the simulated mutant frequency shows a complex dependence on the parameters, the cost function is likely to have multiple local minima. As a consequence, the set of optimal parameters returned by the optim function strongly depend on their initial values. To effectively explore the parameter space, we randomly sampled initial values according to the bounds in table 2 for the parameters and repeated the optimization process until 1,863 converged optimization processes were obtained. Each repetition returned a set of parameters and the corresponding cost value. The values generating the smallest cost among the 1,863 obtained were chosen as optimal parameters and presented in table 2:

$$\hat{\theta }=\underset{\theta }{\text{argmin}}J_{\theta }(x)$$

**Table 2:** Optimized Estimates of the Model Parameters

Parameter	Value	Bounds
*γ*: mutation rate per chromosome per generation	$1.55\times 10^{-3}$	$10^{-10}-10^{-2}$
*μ*: mutation rate per plasmid per generation	$5.09\times 10^{-5}$	$10^{-10}-10^{-2}$
*r*: wild-type plasmid replication rate	1.539	1−5
*R*: mutant plasmid replication rate	3.417	1−5
*S*: base-line fitness	0.780	0.5−1
$\kappa^{*}$: optimal number of plasmids	20	1−40
*κ_T_*: threshold for the number of plasmids	25	1−40

We defined the parameter intervals to ensure that they were wide enough to randomly sample the parameters (table 2). Therefore, a large parameter space was explored. The distributions of parameter value estimates are shown in supplementary figure S7, Supplementary Material online. The chromosomal mutation rate *γ* and the plasmid mutation rate were drawn uniformly on a log_10_ scale from $10^{-10}$ to $10^{-2}$. The replication rates for wild-type *r* and mutant plasmid *R* were set in plausible intervals as [1, 5]. The base-line fitness S∈(0.5,1], since a value ≤0.5 means no growth from one generation to the next. The optimal number of plasmids $\kappa^{*}$ and threshold for the number of plasmids κ_T_ leading to zero probability of surviving were chosen from a set (1,2,…,40).

## Results and Discussion

### Experimental Evolution and Duplex Sequencing

As host, we used recombination and conjugation-deficient DH5*α* (F- *recA1*) *E. coli* cells. Suppression of homologous recombination (*recA1* genotype) minimizes formation of plasmid multimers (Summers 1998). The cells were transformed with pBR322—a classical synthetic cloning vector (Bolivar et al. 1977) without a history of association with DH5α host. This plasmid contains a well understood ColE1 replication origin, has copy number of 15–20 per cell in nutritionally unconstrained conditions (Twigg and Sherratt 1980; Plotka et al. 2017) and carries three protein coding genes: a replication mediator *rom* and two antibiotic-resistance factors represented by a *β*-lactamase and a tetracycline efflux pump. To accelerate adaptive events, we used turbidostat, a device that maintains a constant cell density with no restrictions on the supply of nutrients, as it puts additional selective pressure on host cells to increase their maximum growth rate (Bull et al. 2006; Gresham and Hong 2015). To prevent plasmid loss and emergence of plasmid-free segregants, growth medium was supplemented with tetracycline. We specifically chose tetracycline over ampicillin because *β*-lactamase (an enzyme hydrolyzing–lactam antibiotics such as ampicillin) expressed by pBR322 can diffuse into the medium and provide “collateral” resistance to plasmid-free cells (Vega and Gore 2014). At the same time, pBR322 encoded *tetA(C)* resistance to tetracycline is not enzymatic, it is enabled by actively removing tetracycline from the cell using an efflux pump, and thus cannot be leveraged by plasmid-free cells.

Two turbidostat experiments, each consisting of two replicate runs, were performed: short term (60 h; replicates R1 and R2) and long term (318 h; replicates R6 and R7). At time point zero, the turbidostat was inoculated with an aliquot of overnight DH5α culture transformed with pBR322. Approximately two-third of the volume (∼8 ml) was taken from the incubation vessel of the turbidostat at regularly spaced time points (optical density dips in supplementary fig. S1, Supplementary Material online). For short-term experiment, plasmid DNA was isolated from samples at time points 12, 24, 36, 48, and 60 h. For long-term experiment, plasmid DNA was isolated from samples at time points 72, 84, 108, 156, 240, and 318 h. Plasmid DNA was subjected to duplex sequencing without any additional manipulation. Sampling a large fraction of bacterial culture allowed us to perform thorough population sampling. In each case, both replicates were sequenced for a total of 24 duplex-sequencing data sets. We also performed conventional (nonduplex) high-depth sequencing of the host genome before (naïve) and after (evolved) the experiments.

### Emergence of Variation Followed by Crash

**Fig. 1.**
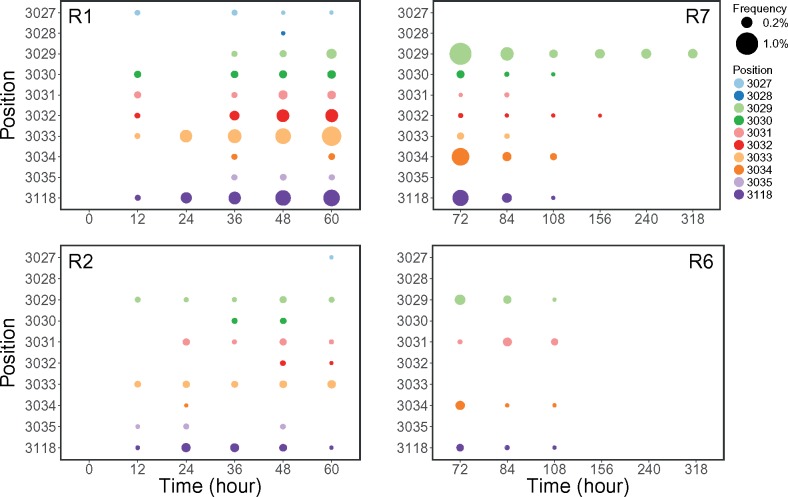
—Locations and frequencies of variants detected at bases 3,027–3,035 and 3,118 on pBR322 with duplex sequencing. R1 and R2 denote the two replicates of short turbidostat run. R6 and R7 denote the two replicates of long turbidostat run. Positions are projected to the *y* axis, and colored individually. Sizes of the closed circles are proportional to the allele frequencies of corresponding variants.

Analysis of single-nucleotide variants (SNVs) revealed by duplex sequencing (fig. 1 and supplementary table S1, Supplementary Material online) identified two classes of changes. First, there were low-frequency variants that do not form a stable trajectory across the time points—they are present in one time point but disappear in the next. For example, the initial, monomorphic, samples (time point zero, short-term experiments, replicates R1 and R2) contained five SNVs supported by a single duplex family each (supplementary table S1, Supplementary Material online). We call such positions flickering sites, which likely represent neutral variants lost due to genetic drift. Flickering sites are present in most (but not all) time points, and the vast majority of them are supported by only one duplex family. The second group of variants is represented by likely adaptive SNVs that persist through the time points. None of them appear in the initial sample, and their frequencies increase initially (short-term experiment) and start to drop off as the experiment progresses (long-term experiment; fig. 1) so that, at the terminal point of one of the replicates of the long-term experiment, pBR322 appears to reverse completely to the wild-type. These SNVs cluster exclusively in two regions within the ColE1 replication origin of pBR322: positions 3,027–3,035 and 3,118. In total, all experiments yielded 145 SNVs. The mutational spectrum (supplementary fig. S2, Supplementary Material online) showed a transition-to-transversion ratio of 1:1.9, similar to that of the long-term evolution experiment (Wielgoss et al. 2011). One insertion and two deletions were identified in total (supplementary table S1, Supplementary Material online). The insertion was located at the replication origin, and was observed only in replicate R7 of the long experiment. One deletion that was located outside the origin was found in multiple turbidostat runs. The other deletion was within the origin, and was only observed in the R1 replicate of the short experiment. Indels did not exhibit any frequency fluctuation, which could be due to the rate of indel mutations being generally lower than that of base-pair mutations (Drake 1991; Lee et al. 2012; Jee et al. 2016).

Because the distance between SNVs located within the replication origin is shorter than the length of the duplex reads, we examined our data for evidence of linkage. No linkage between any SNVs was observed. This is not unexpected because given the *E. coli* mutation rate is $∼10^{-10}-10^{-9}$ per nucleotide per generation (Drake 1991; Wielgoss et al. 2011; Lee et al. 2012; Jee et al. 2016) and the highest frequency attained by these SNVs is ∼0.9% it is highly unlikely to have two mutation events within a single molecule.

### Variants Are Restricted to Copy Number Control Elements

The mechanism controlling pBR322 (ColE1) replication has been extensively studied by Tomizawa and coworkers (Eguchi et al. 1991). Two complementary RNA molecules are encoded within the *ori* region: RNAI and RNAII. RNAII, a *cis*-acting activator, is first transcribed and hybridizes with plasmid DNA to form a primer precursor for DNA replication. The other molecule—RNAI, a *trans*-acting inhibitor—is transcribed from the complementary strand in the opposite direction (supplementary fig. S3*A*, Supplementary Material online). RNAI inhibits the formation of RNAII–DNA complexes and resulting replication primer, by initially interacting with RNAII via three loop regions and subsequently forming a stable duplex assisted by a plasmid encoded Rom protein. Substitutions within region 3,027–3,035 (supplementary fig. S3*A* and *B*, Supplementary Material online) were reported to increase pBR322 copy number (Davison 1984). This interval represents loop II’ of RNAI. Meanwhile, base 3,118 corresponds to the −35 promoter region of RNAII (supplementary fig. S3*C*, Supplementary Material online). Changes at this base increase the copy number as well (Castagnoli et al. 1985).

### Evolved Cells Have Higher Fitness Conferred by Chromosomal Mutations

At each collection point, we sampled two-third of volume from the incubation vessel, which was immediately topped-off with sterile medium diluting the remaining cells and causing optical density dips in supplementary figure S1, Supplementary Material online. In each case, OD_600_ values quickly returned to the 0.8 threshold. The speed of this recovery can be used as a relative fitness estimate: the steeper the slope, the fitter the cells. This approach demonstrated consistent fitness increases over the course of turbidostat runs (supplementary fig. S4, Supplementary Material online; black dots). Contrasting the fitness of terminal clones from replicates R6 and R7 against the clones from the initial time point zero stocks confirmed this pattern (supplementary fig. S4, Supplementary Material online; red triangles).

The fact that pBR322 has completely reverted to wild-type in R6 (supplementary table S1, Supplementary Material online) and was on the way to purge all variation in R7 suggests that the increase of fitness can be attributed to changes within the bacterial chromosome. To confirm this, we cured evolved strains from pBR322 and compared their growth rates in LB medium without antibiotic—a proxy for overall fitness. Results of this experiment are given in table 1. Plasmid-containing time point zero inoculates R6-S0 and R7-S0 experience a small decrease in fitness relative to plasmid-free cells. However, the evolved strain R6-S27 effectively more than doubles its fitness, while R7-S27 exhibits only a mild increase. Curing plasmids gives only small fitness boost for R6-S27 but simultaneously doubles the fitness of R7-S27. Thus, both R6-S27 and R7-S27 decreased the cost of plasmid carriage but this charge is the most pronounced in the former where there is essentially no difference in the growth rate with or without plasmid.

To determine the nature of adaptive changes within the bacterial chromosome, we performed whole-genome sequencing of the host genome before and after the experiment. Two nonsynonymous substitutions were identified in both replicates: a T-to-A transversion in gene *treB* (Val112Glu) and a C-to-T transition in *recA* (Asp161Gly). The allele frequencies at these two sites differed drastically between replicates. For *treB*, they were 92% and 39% whereas for *recA* 91% and 7% in R6 and R7, respectively. This effectively reverses the RecA1 genotype back to RecA in R6. R7 is delayed but follows the same trajectory.

These results raised a question on how to explain the discrepancy between growth rates of plasmid-containing and cured R6 and R7 isolates listed in table 1: why is the growth rate of plasmid containing R6-S27 twice that of R7-S27, while cured R6-S27 and R7-S27 have largely identical growth rates? The answer is in the fact that for plasmid-containing isolates the growth rate was measured for the population of cells, while for plasmid-free growth rates are for individual clones picked as single colonies (supplementary fig. S5, Supplementary Material online, explains the curing process). Because *treB* and *recA* mutations are present in R7-S27 (albeit in much lower frequencies than in R6-S27) plating process effectively selected for R7-S27 cells that contain both mutations as they grow faster. This was confirmed by Sanger sequencing as shown in supplementary figure S6, Supplementary Material online. The fact that these mutations increase growth rate with or without plasmid indicate that they are likely involved in adaptation to the growth conditions rather than to the presence of the plasmid.

The product of the *treB* gene transports trehalose into the cell as trehalose 6-phosphate, which is further converted to glucose 6-phosphate and glucose (Klein et al. 1995). In addition to its role as a carbon source, intracellular trehalose can act as an osmoprotectant at high osmolarity (Boos et al. 1990; Rimmele and Boos 1994; Park et al. 2016). The T-to-A transversion in *treB* observed in our work has been suggested to be a consequence of adaptation of *E. coli* to LB medium and has been identified in three parallel populations in two recent mutation accumulation experiments (Blaby et al. 2012; Behringer et al. 2018).

The RecA1 → RecA reversal in replicate R6 is noteworthy, as it is close to fixation (frequency of 91%) and reinstates recombination (Sancar et al. 1980; Kawashima et al. 1984; Bryant 1988). Coincidentally, the same replicate R6 of the long experiment purges plasmid variation completely at the end of sampling, while the other replicate, R7, which did not yet experience complete RecA1 → RecA reversal continues to harbor lingering variation within the plasmid. Recombination has been shown to accelerate adaptation in yeast populations by combining beneficial mutations from different backgrounds and by separating deleterious mutations apart (McDonald et al. 2016). However, none of the variants we observed are linked, thus, it is unlikely if RecA has any direct effect on the pattern observed here.

### Variants Affecting Copy Number Do Not Easily Fix

The observations we described thus far suggest the following scenario. A naïve host is transformed with a plasmid conferring antibiotic resistance. A crowded turbidostat environment promotes competition among cells for shortening generation time. In the presence of tetracycline, an antibiotic interfering with protein synthesis by blocking the interaction between aminoacyl-tRNAs and ribosomes (Chopra and Roberts 2001), bacterial cells can potentially shorten the generation time by increasing the rate with which the drug is removed from the cell. This can be accomplished by increasing the expression of tetracycline-resistance gene encoded by pBR322. This, in turn, can be achieved by either 1) increasing the transcription and/or translation output of the gene or 2) increasing the plasmid’s copy number, which effectively multiplies the number of tetracycline-resistance genes per cell. The clustering of all plasmid substitutions observed here within RNAI/II genes points to the latter option. After some time, a mutation in RNAI/II locus occurs during plasmid replication. At this point, the cell becomes heteroplasmic—its plasmid population is no longer homogeneous. Because RNAI can act in *trans*, a single mutated plasmid can potentially produce RNAI transcripts affecting replication of other wild-type plasmids within the cells thereby increasing their copy number. Depending on timing of the mutation event relative to the stage of cell cycle there may be just one or multiple plasmids carrying the change (e.g., depending on whether the mutated plasmid has been replicated right before the division or not). Upon cell division, mutated plasmids will be randomly partitioned across the daughter cells. As a result, three outcomes are possible for each daughter cell to receive: 1) no mutated plasmids, 2) a mix of the two, or 3) all mutated copies. The first possibility simply reverts cells to wild-type. An increase in plasmid copy number incurs additional metabolic burden on the cell while the simultaneous increase in the efflux pump expression can also be toxic due to loss of membrane potential (Eckert and Beck 1989). This makes the third scenario likely deleterious. Yet, the second outcome is the most interesting. It is possible to envision a situation in which a heteroplasmic cell with a particular “perfect” ratio of mutated/wild-type plasmids finds a balance between combined deleterious effect of the increased copy number and the benefit or rapid tetracycline removal. The difficulty is that this balance is likely to be broken at every cell division due to stochastic plasmid segregation. As a result, one can expect to have a relatively small number of “perfectly balanced” cells at any given time. This slows the spread of mutations through the population of cells. At the same time, if a beneficial mutation occurs on chromosome, then its stable inheritance through cell doubling will quickly fix it in the population (barring outcomes of clonal interference with other large effect chromosomal mutations). Given the low frequency of plasmid mutations observed here, it is highly unlikely that such a chromosomal mutation will arise on background of plasmid mutations. In other words, cells containing the chromosomal mutation will likely contain only wild-type plasmids. Therefore, the spread of chromosomal change to fixation will obliterate plasmid variation in the short term—the exact dynamics observed in our experiment.

To put this logic into quantitative framework and to allow forecasting, let us assume that the dynamics of mutant and wild-type plasmids in cells from generation to generation follows three iterating stages:

1. *division* of cells into two daughter cells;
2. *replication* that involves increase (replication) in the number of wild-type and mutant plasmids in each cell as well as mutation from wild-type to mutant plasmids or mutation on a chromosome;
3. *selection* based on the number of plasmids a cell contains or the presence of a chromosomal mutation.

We model this using seven parameters *γ*, *μ*, *r*, *R*, *S*, $\kappa^{*}$, and *κ_T_* as described in Materials and Methods. Here, *γ* represents the probability that a chromosomal mutation will arise in a cell that does not have a chromosomal mutation during replication, *μ* represents the probability that a wild-type plasmid will change to a mutant plasmid during replication, *r* and *R* are the respective replication rates for wild-type and mutant plasmids during replication, S∈(0.5,1] is a base-line fitness value for cells with one plasmid and no chromosomal mutation, $\kappa^{*}\in \{1,2,\ldots ,\kappa_{T}-1\}$ is the optimal number of plasmids, $\kappa_{T}\in \{21,22,\ldots ,40\}$ is a threshold for the number of plasmids per cell such that cells with at least *κ_T_* plasmids have zero probability of surviving due to associated metabolic cost and overexpression of the efflux pump (the range of values for $\kappa^{*}$ and *κ_T_* parameters are based on pBR322 copy number in *E. coli* cells).

**Fig. 2.**
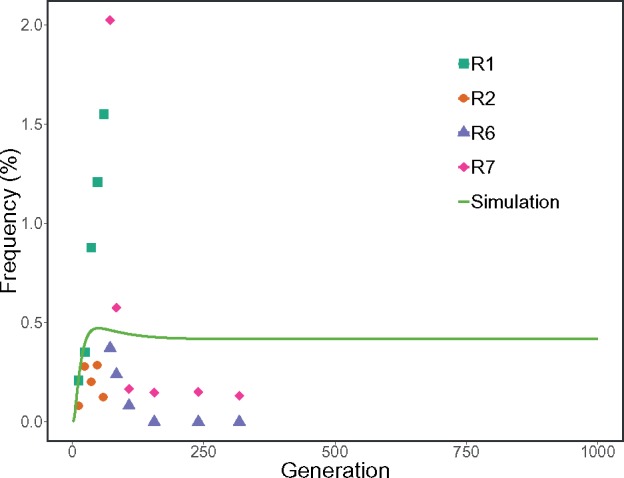
—A combination of empirical (R1, R2, R6, and R7) and predicted (simulation) mutant plasmid frequencies. The simulation was performed with parameters obtained by numerical optimization (see Materials and Methods). Different turbidostat runs are colored, respectively. *y* axis shows the sum of frequencies at bases 3,027–3,035 and 3,118. *x* axis represents generations. The *x* axis here using generation as the unit corresponds to the *x* axis using time in hours in figure 1. This conversion was accomplished by assuming that the generation time was constantly 60 min. The empirical data were collected for 318 generations.

At each sequenced time point, we can directly estimate the fraction of mutants in the population as the sum of alternative allele frequencies at sites 3,027–3,035 and 3,118. This corresponds to *β_t_* in the model and allows fitting the model to the empirical data as described in Materials and Methods. Briefly, we estimated *β_t_* from the observed allele frequencies at each sequenced point. We then set biologically plausible intervals of possible values for the *γ*, *μ*, *r*, *R*, *S*, *κ_T_*, and $\kappa^{*}$ parameters, and employed numerical optimization to infer a set of parameters minimizing the differences between simulated and empirical values of *β_t_*. Resulting optimized values of the seven parameters are given in table 2. Finally, we used the estimated parameter values to simulate the behavior of plasmid alleles for an extended period of time (fig. 2). There is a sharp increase in the frequency of plasmid mutations in the first few generations, suggesting the benefit of carrying more plasmid copies. A slow decrease was then observed due to expansion of chromosomal mutations bearing lineages as well as the deleterious effect of carrying more plasmid than the optimal number $\kappa^{*}$. As the chromosomal mutation becomes fixed, the frequency of plasmid alleles reaches a plateau below 0.5%—they linger but never fix. This plasmid allele frequency trajectory based on our optimal model parameters also does not recapitulate the sharp decline and loss of mutant plasmid alleles with increasing number of generations. This balance of plasmid allele frequency is likely due to the fitness model that we have imposed (see Materials and Methods), and other fitness models may better fit the sampled empirical frequencies. However, our goal with this model was to demonstrate that plasmids containing mutations affecting copy number may not reach fixation under inferred biologically plausible model parameters (table 2).

### Plasmid Copy Number Control Is Robust against Adaptive Changes

In this experiment, we transformed naïve *E. coli* cells with a plasmid carrying an antibiotic-resistance gene and forced them to adapt to a turbidostat environment. Sequence variants revealed by duplex sequencing showed that cells initially responded by allowing changes to the replication control machinery of pBR322. The frequencies of these changes never reached 1% and declined toward the end of the experiment, while two chromosomal mutations appeared to be on the path to fixation. We hypothesized that a seemingly straightforward approach to shortening generation time by increasing the plasmid copy number and corresponding increase in the number of tetracycline efflux pumps is in fact quite difficult to achieve. This is because cells need to balance the benefit of antibiotic removal with the metabolic burden of extra plasmid copies and deleterious effects associated with the overexpression of the efflux pump. We devised a model describing this behavior, estimated its parameters, and simulated the behavior of mutations changing copy number for an extended period. After initial increase, the mutation frequencies stabilized and remained <0.5%. These results indicate that plasmid copy number is a stable trait resistant to change. Results from other groups support this conclusion. In a recent work, San Millan et al. (2016) adopted *E. coli* cells containing a plasmid carrying *bla*_TEM-1_-lactamase to progressively increasing concentrations of ceftazidime. These authors report two types of changes. First, a *bla*_TEM-1_ mutation known to confer ceftazidime resistance reached fixation. Second, similarly to our data, they also observed mutations in the RNAI gene and detected the corresponding increase in the copy number. However, mutations affecting copy number never reached fixation and appeared in detectable frequencies only after ceftazidime concentration was raised to 32× of the minimal inhibitory concentration (MIC; for comparison tetracycline concentration in our experiment was 30 µg/ml, which is ∼7.5× MIC for this drug). These results imply that selection may act on mutations affecting copy number differently than it treats other mutations. A mutation that enables a plasmid to replicate at a higher rate than its wild-type counterparts has a higher chance of persisting across generations. At the same time, increased copy number puts a higher burden on host cells threatening their persistence in the population (Paulsson 2002). Thus, from the standpoint of the host, mutations appear in individual plasmid molecules while selection acts on an assemblage of all plasmids in a given cell (Bergstrom and Pritchard 1998). For a “conventional” adaptive mutation such as in the *bla*_TEM-1_ example mentioned above, selection will favor a cell where all plasmids carry the R164S amino acid replacement since this enables maximum TEM-1 resistance toward ceftazidime. Yet, for mutations that affect copy number, the situation is drastically different. All plasmid mutations encountered in this study affect RNAI (sites 3,027–3,035) or the promoter of RNAII (site 3,118). RNAI inhibits replication initiation in *trans* (Tomizawa and Itoh 1981): an RNAI mutation is public and affects all plasmids within a given cell whether mutated or not. RNAII is a replication primer precursor that is *cis*-acting: it only affects plasmid copy from which it is transcribed (Eguchi et al. 1991). This ensemble of *trans*-acting inhibitor and *cis*-activator are aided by the Rom protein that promotes formation of a stable RNAI–RNAII complex (Tomizawa and Som 1984). This system maintains optimal copy numbers balancing between two undesirable extremes: segregational loss of plasmids if copy number is low or a runaway replication imposing prohibitive burden on the host cells (Paulsson et al. 1998). This balancing act appears to be robust against fluctuations even, as is the case of our study, if they are caused by adaptive changes (Paulsson and Ehrenberg 2001). Our results suggest that there is a strong incentive not to alter copy number even if it can provide a degree of selective advantage. This incentive is likely rooted in the complex interplay between mutated and wild-type plasmids constrained within a single cell and underscores the importance of understanding of intracellular plasmid variability.

### Challenges and Future Experiments

Although our model captures the underlying dynamics well, there are a number of discrepancies and unknowns. If it is so difficult to maintain a balance between the benefit of increased copy number and related metabolic burden, then how does the mutation we observed reach even these low but still detectable frequencies? Why in our experiment do the frequencies of plasmid mutations fall rapidly after the fixation of chromosomal changes while they persist in our best fit model? Answering these questions will require assessing the validity of our assumptions and considering new experiments. Mutation frequencies shown in figure 2 are estimated population frequencies—a fraction of all plasmids across all sampled cells that carry a particular mutation. If these were chromosomal mutations, then they will be locked into a clonal interference competition. Yet, these mutations are constrained within individual cell lineages and never actually interact with each other. This raises the question of whether they are fixed—all plasmids within a cell carrying a mutation—or exist in heteroplasmic state as was shown in other systems (Santos-Lopez et al. 2017; Rodriguez-Beltran et al. 2018). This question cannot be answered without an experiment in which individual lineages are explicitly tracked using molecular barcodes. Another important challenge of our experimental design is the fact that our study was performed in a well-mixed liquid culture where any cell competes against the entire population. The situation will likely be quite different on solid medium or in biofilms, where outcomes of selection are determined by immediate neighborhood rather than the entire population. Finally, here, we assume random partitioning of plasmids where every plasmid has an equal chance of segregating with either of the daughter copies. A series of recent reports challenge this assumption and point to uneven segregation of plasmids at cell division (Hsu and Chang 2019; Münch et al. 2019). Furthermore, although our experiment was performed in cells deficient in homologous recombination, we cannot rule out formation of plasmid dimers and multimers. Multimers effectively decrease the number of segregating units and therefore affect partitioning dynamics (Chiang and Bremer 1988; Summers 1998). 

## Data Availability

Jupyter notebooks and data to perform the parameter optimization (table 2) and simulation shown in figure 2 can be found at https://github.com/hanmei5191/Plasmid_heteroplasmy, last accessed September 19, 2019.

## Supplementary Material

Supplementary data are available at *Genome Biology and Evolution* online.

## Supplementary Material

evz197_Supplementary_Data

## Notes

**Data deposition:** This project has been deposited at Short Read Archive (SRA) at the National center for Biotechnology Information (NCBI) under the accession PRJNA485503.
