# Supplementary figures and images for: A High-Resolution View of Adaptive Event Dynamics in a Plasmid
Source: Genome Biol Evol. 2019 Sep 18;11(10):3022–34. doi: 10.1093/gbe/evz197 (PMC6827461; doi:10.1093/gbe/evz197)

**R1**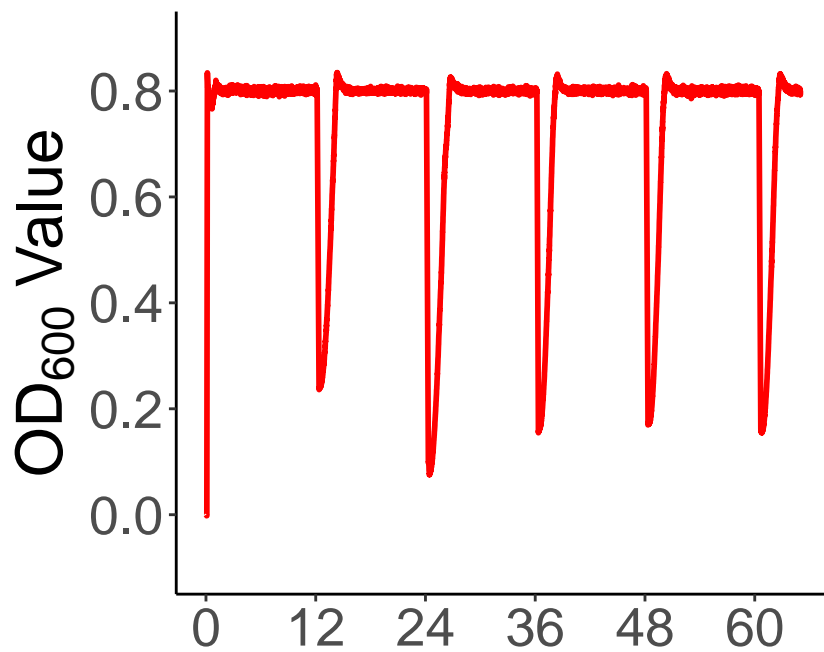**R6**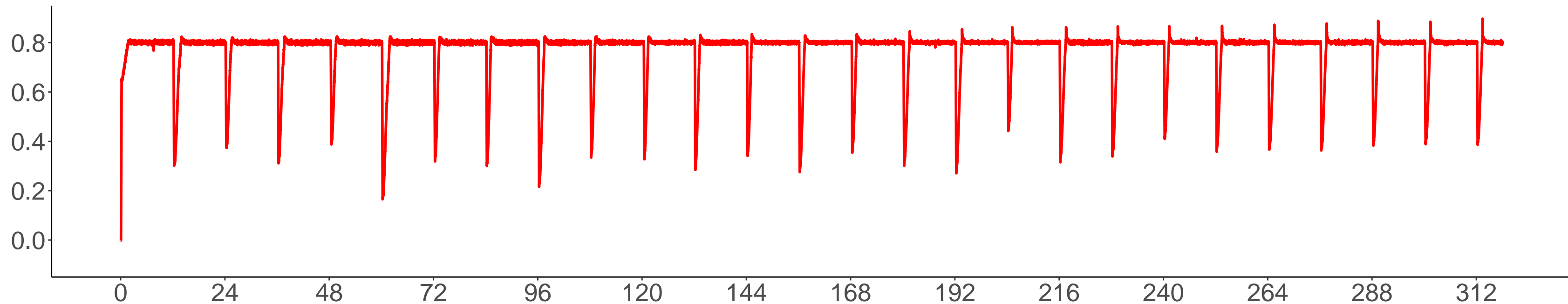**R2**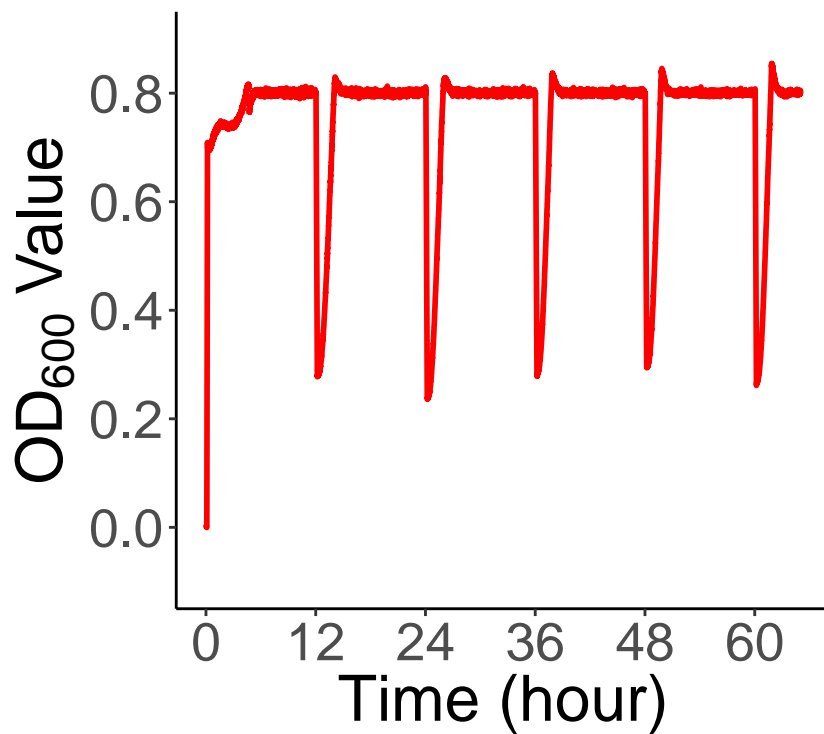**R7**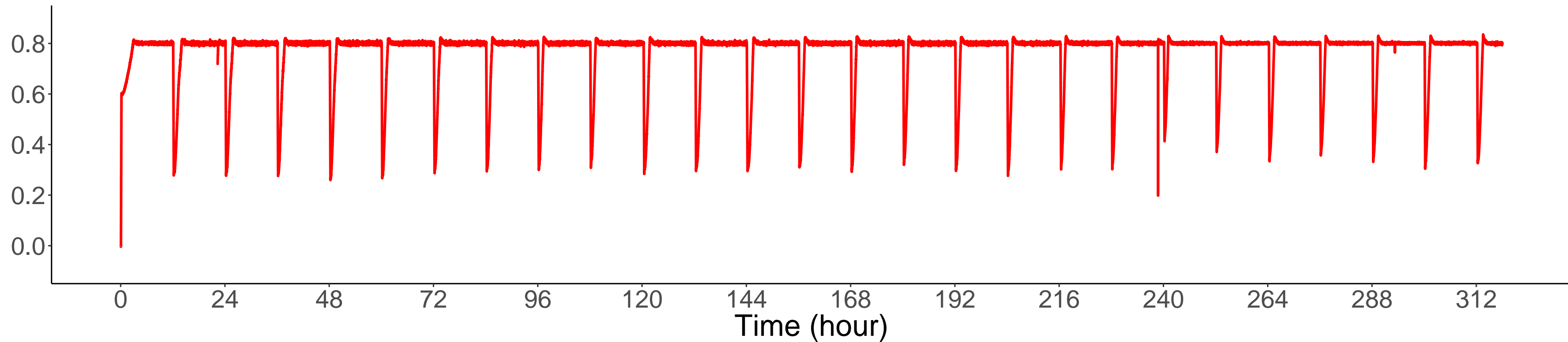

Supplement: evz197_Supplementary_Data [file evz197_supplementary_data.zip › Figure_S1.pdf]

# Mutational spectrum

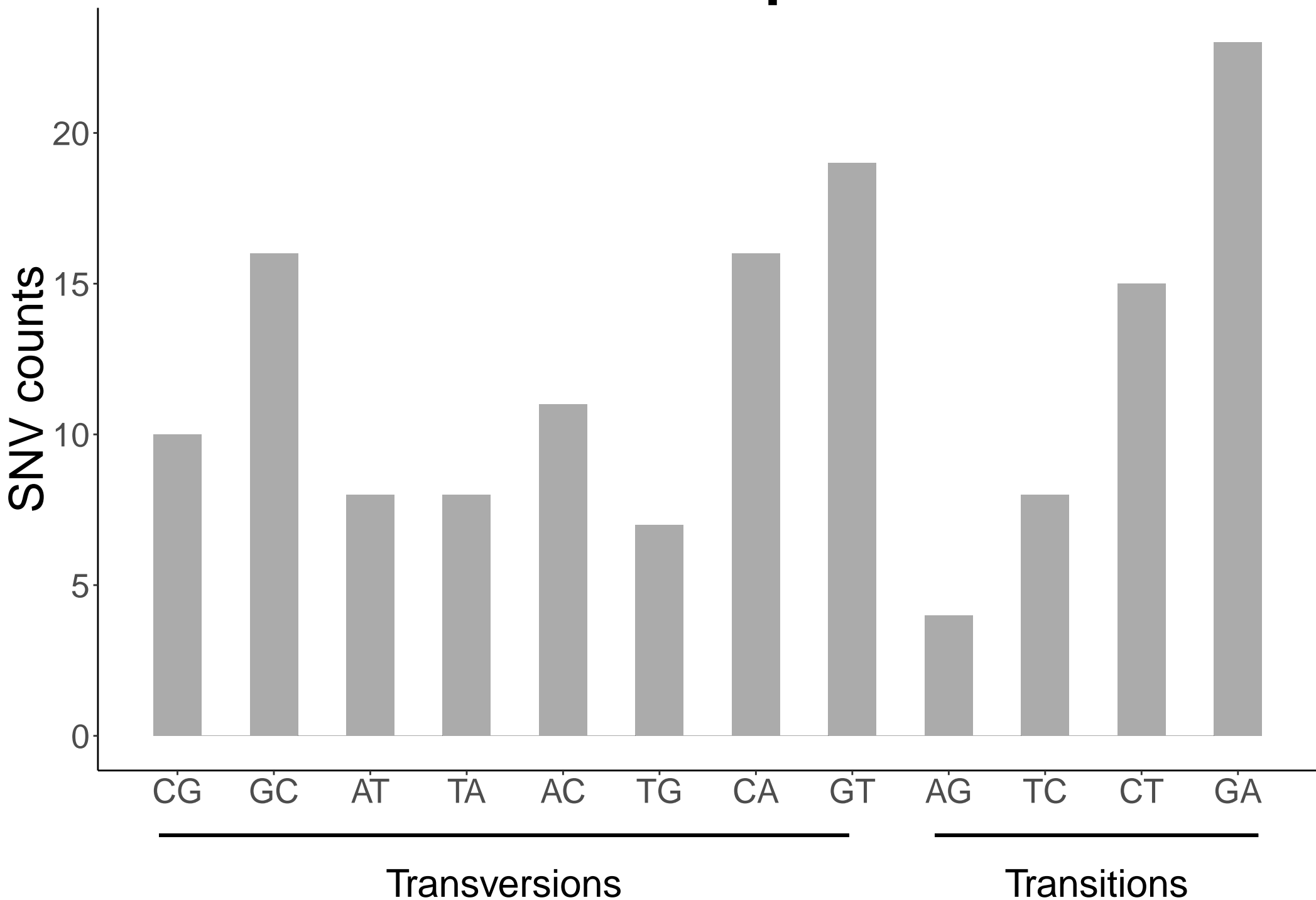

Supplement: evz197_Supplementary_Data [file evz197_supplementary_data.zip › Figure_S2.pdf]

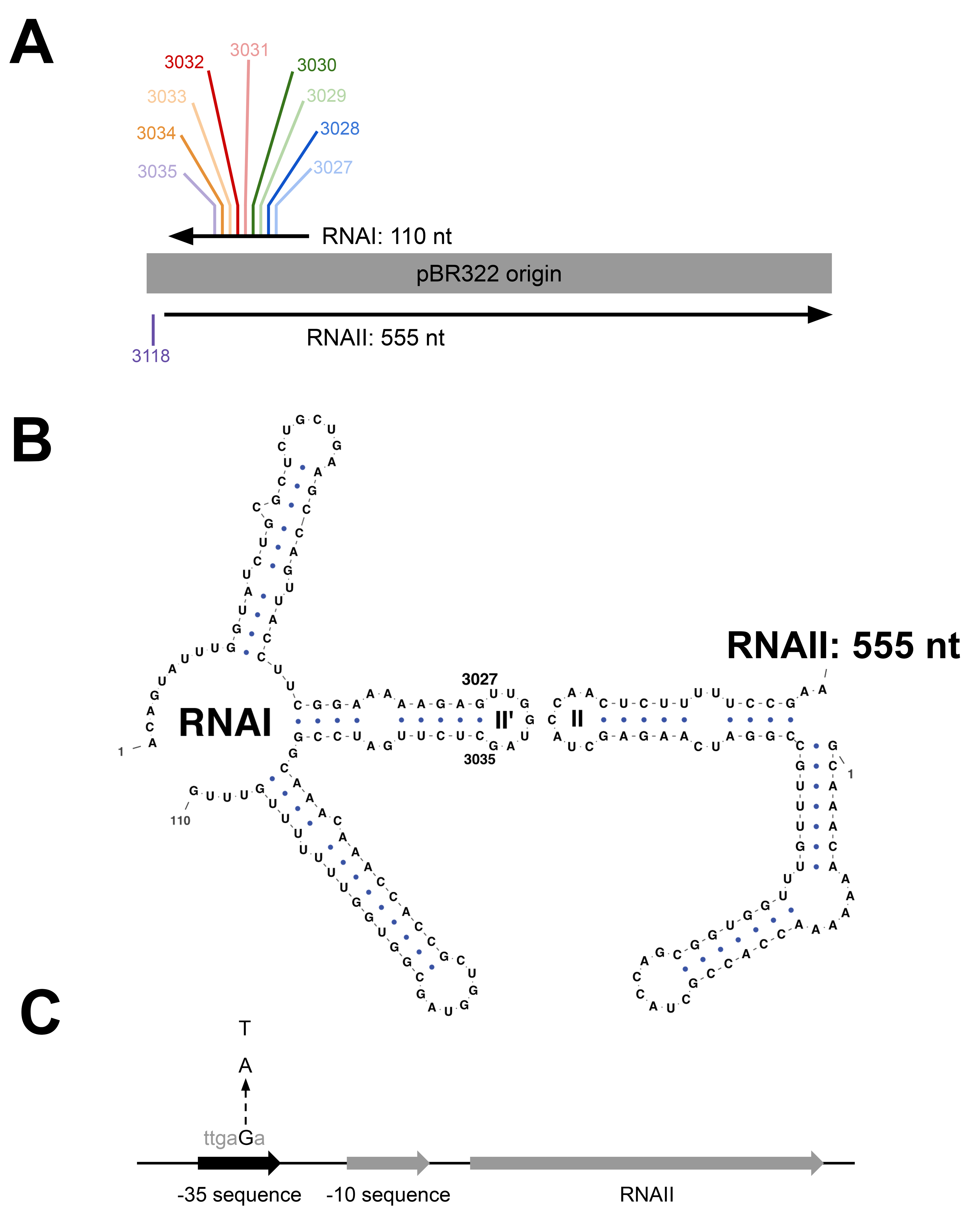

Supplement: evz197_Supplementary_Data [file evz197_supplementary_data.zip › Figure_S3.tif]

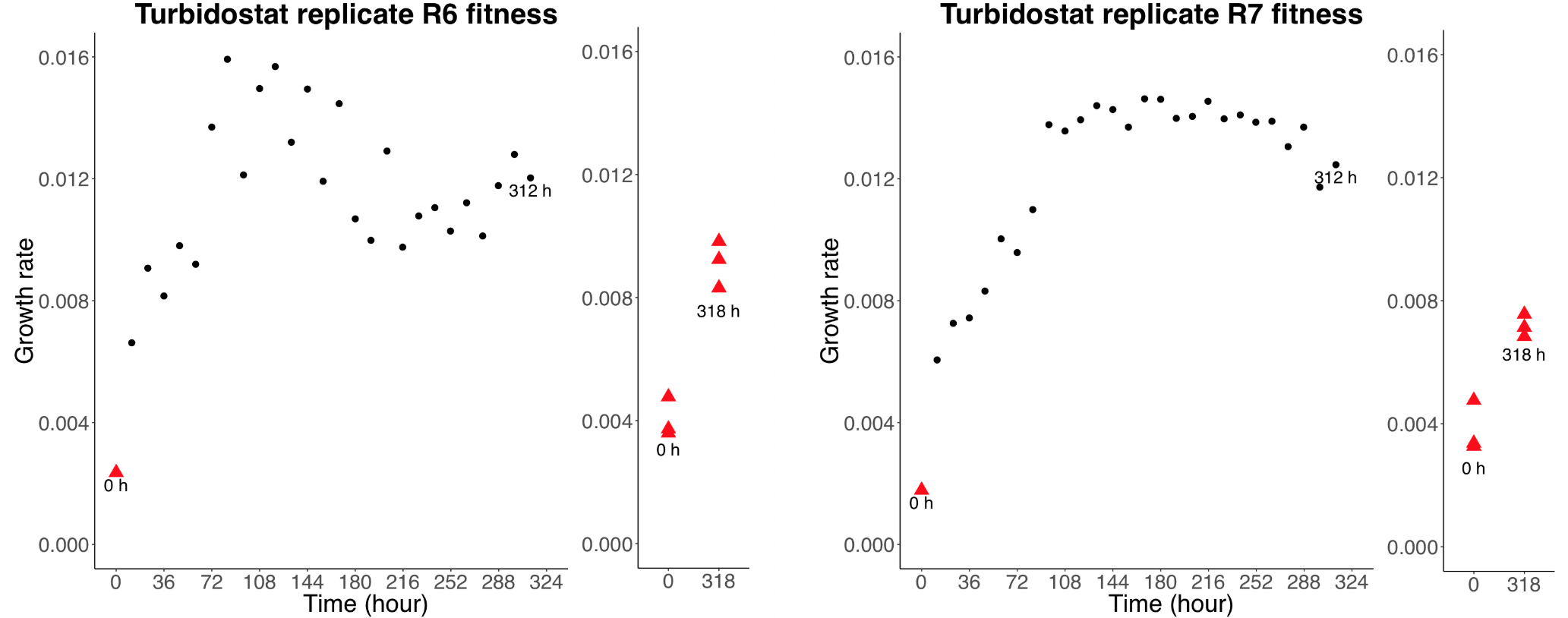

Supplement: evz197_Supplementary_Data [file evz197_supplementary_data.zip › Figure_S4.png]

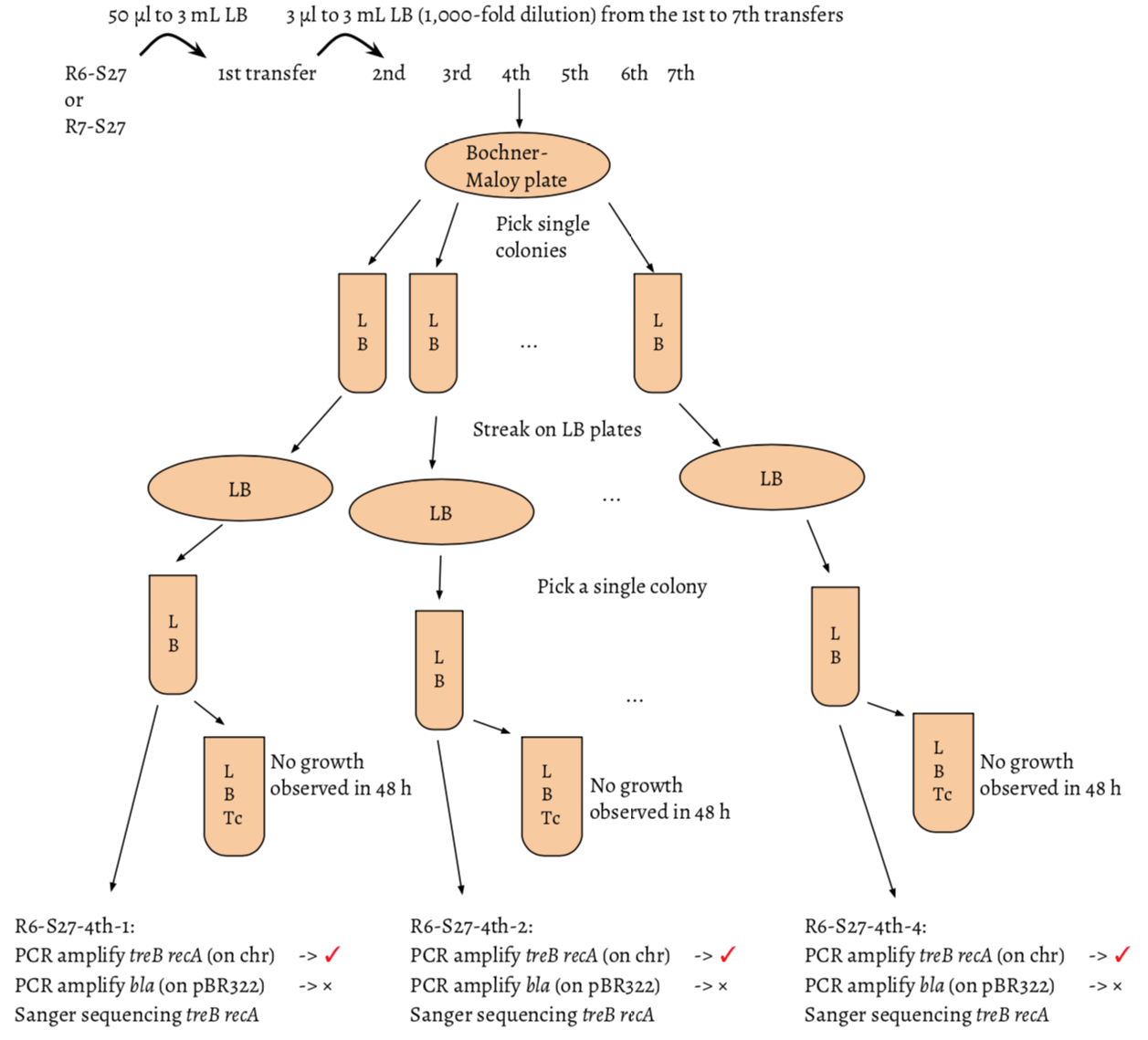

Supplement: evz197_Supplementary_Data [file evz197_supplementary_data.zip › Figure_S5.png]

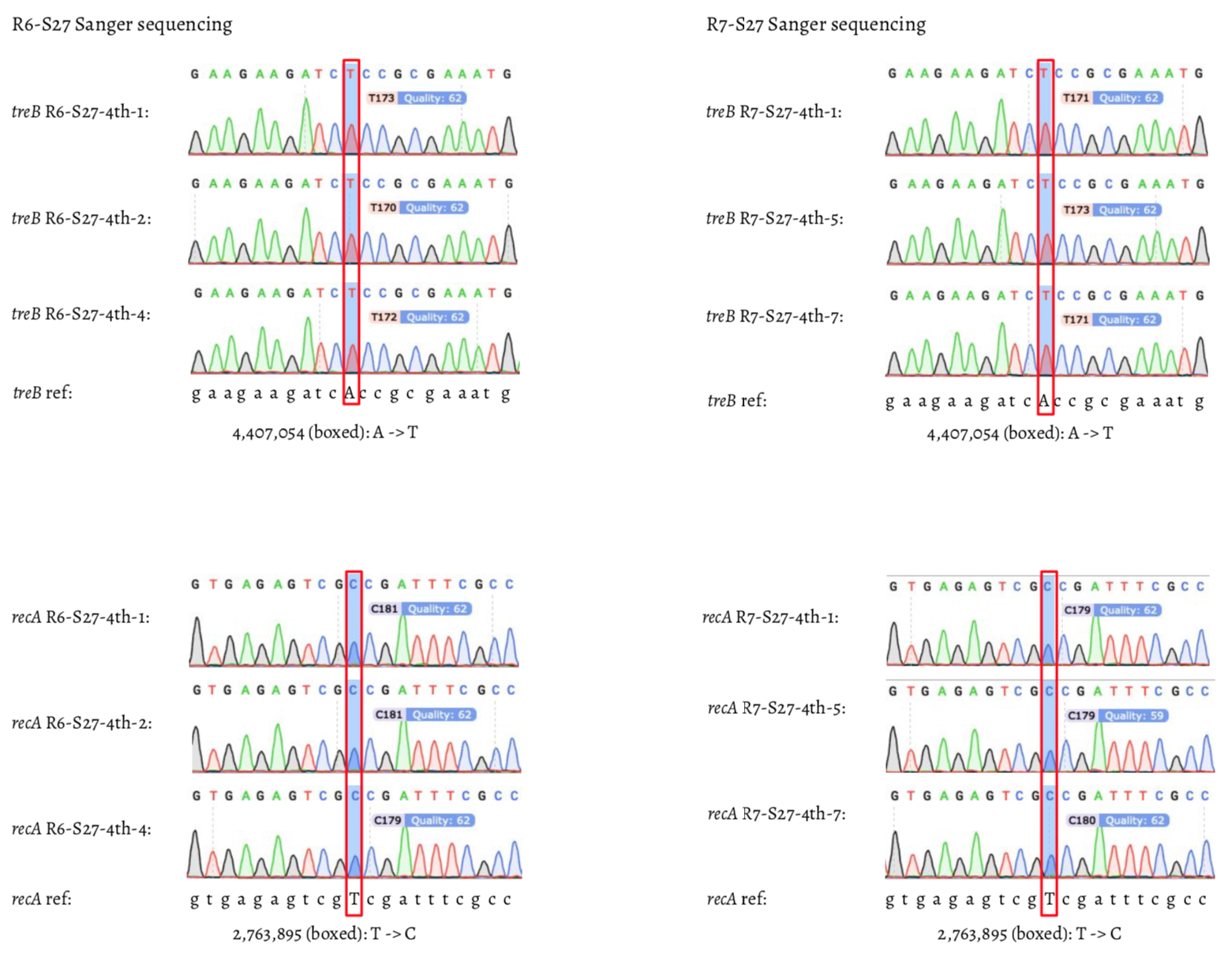

Supplement: evz197_Supplementary_Data [file evz197_supplementary_data.zip › Figure_S6.png]

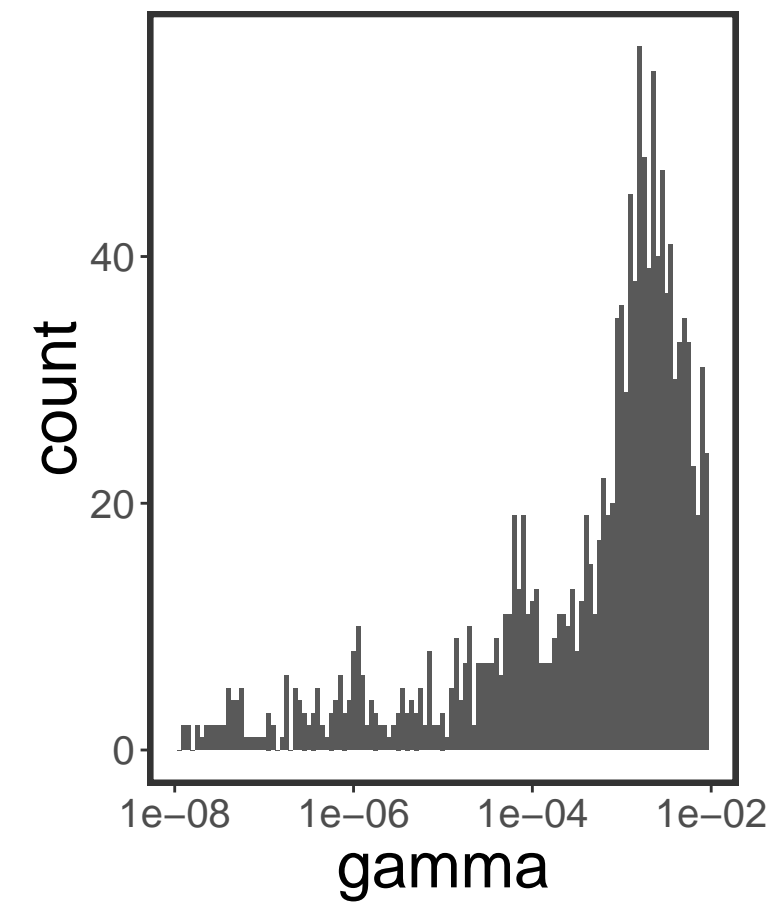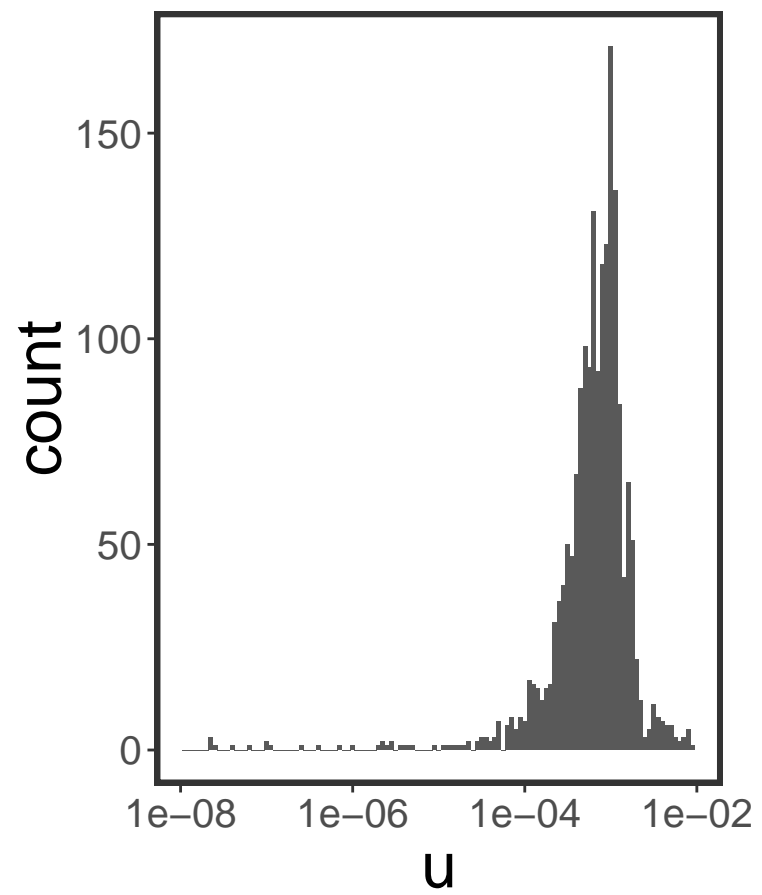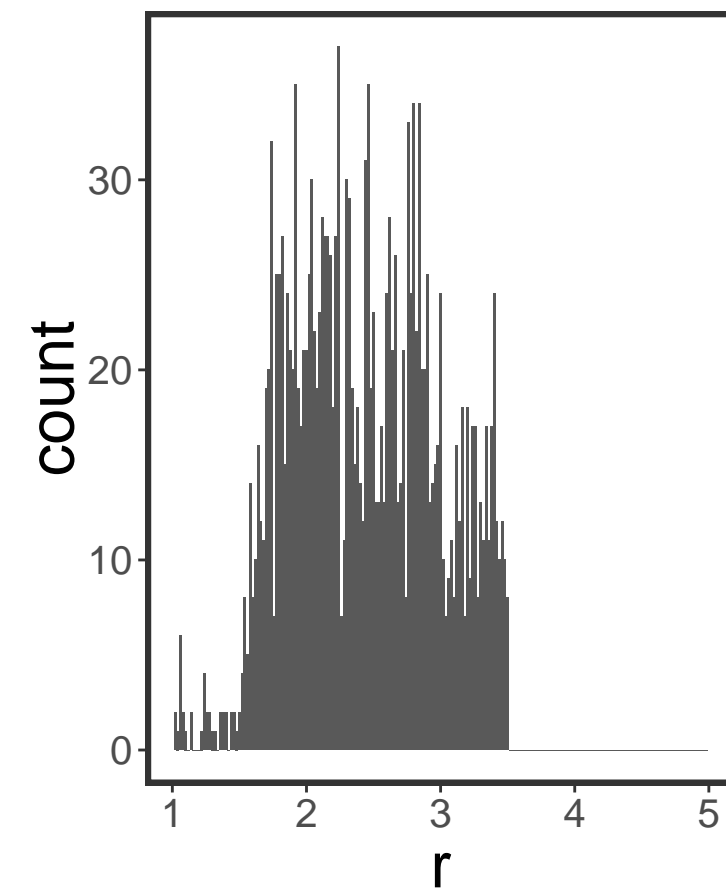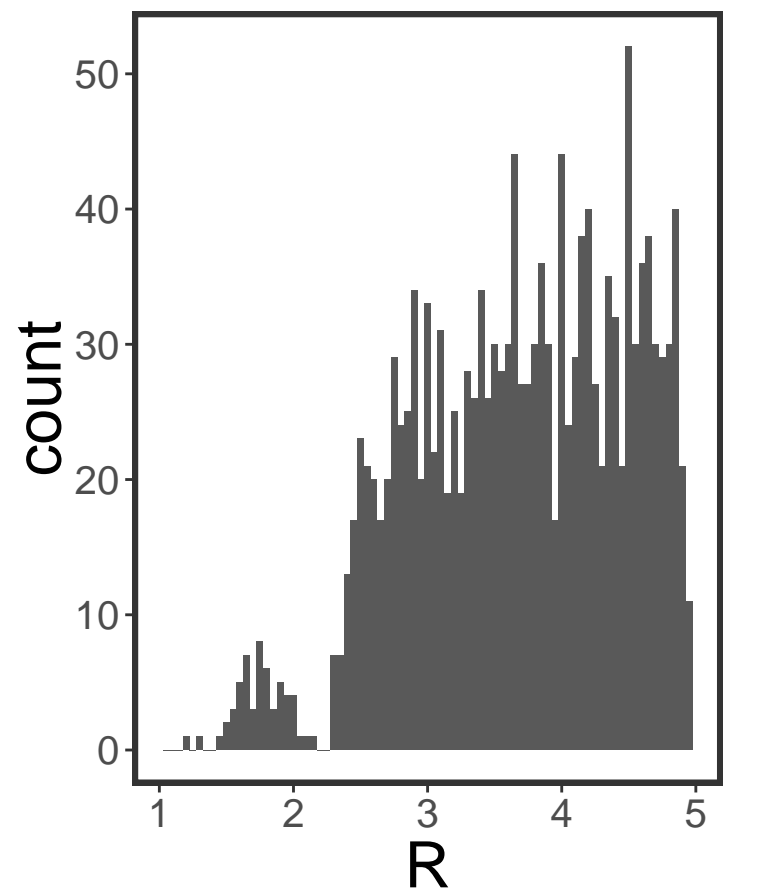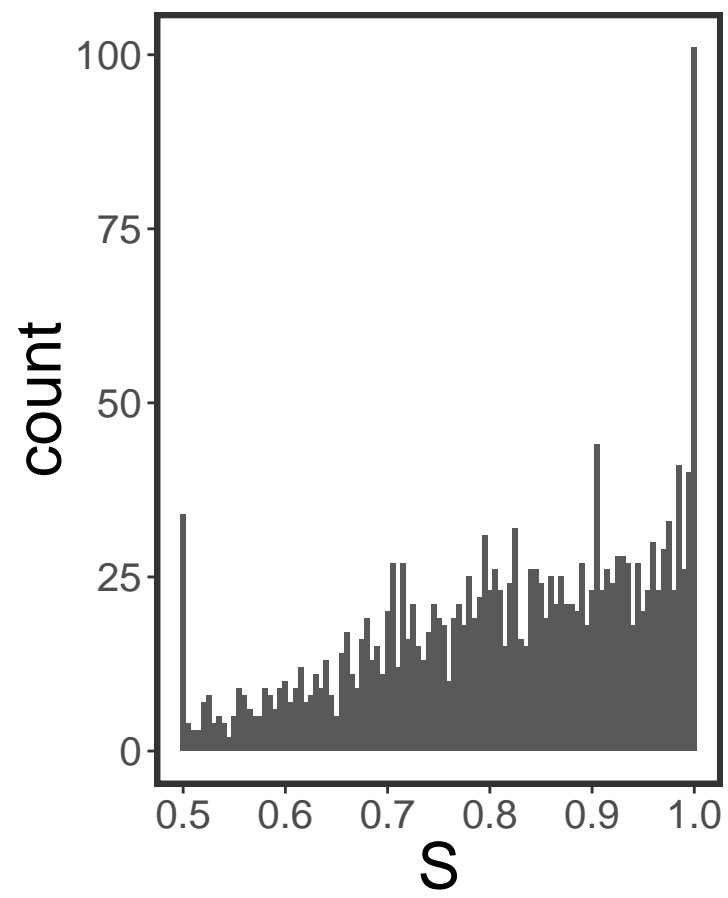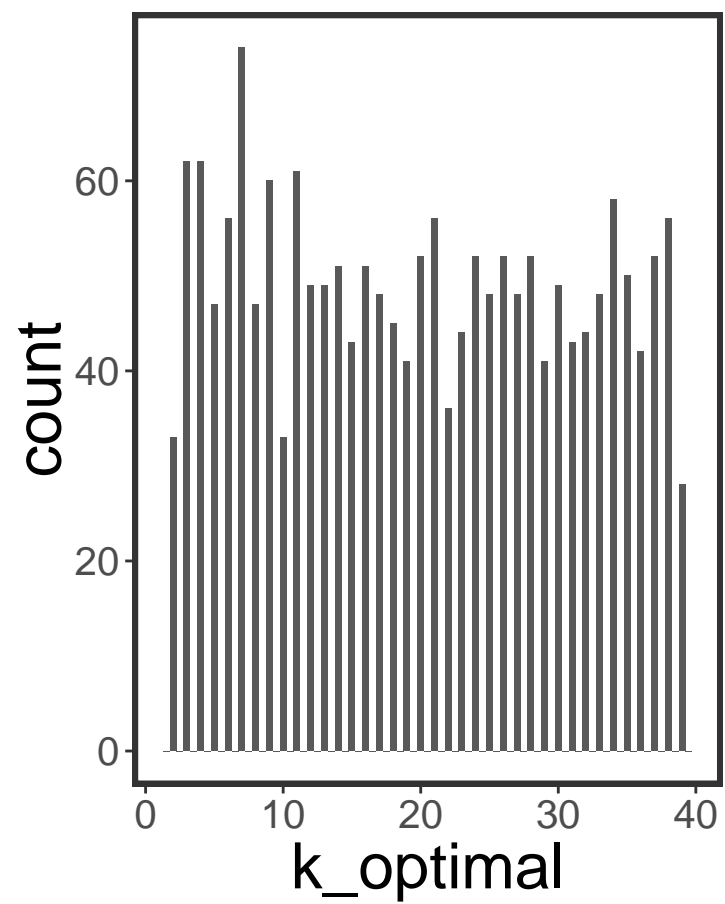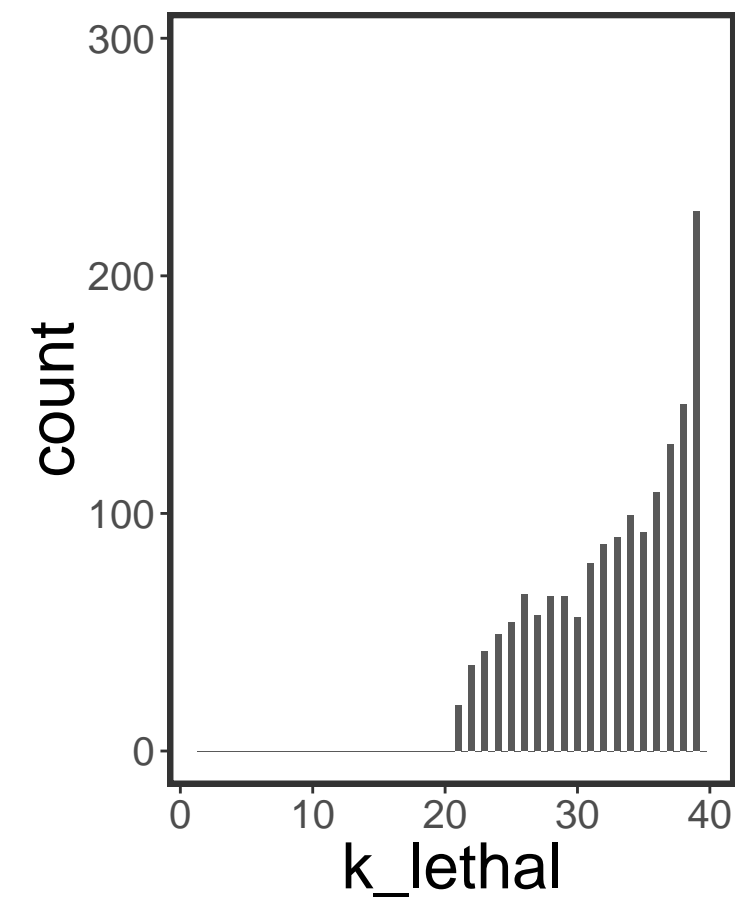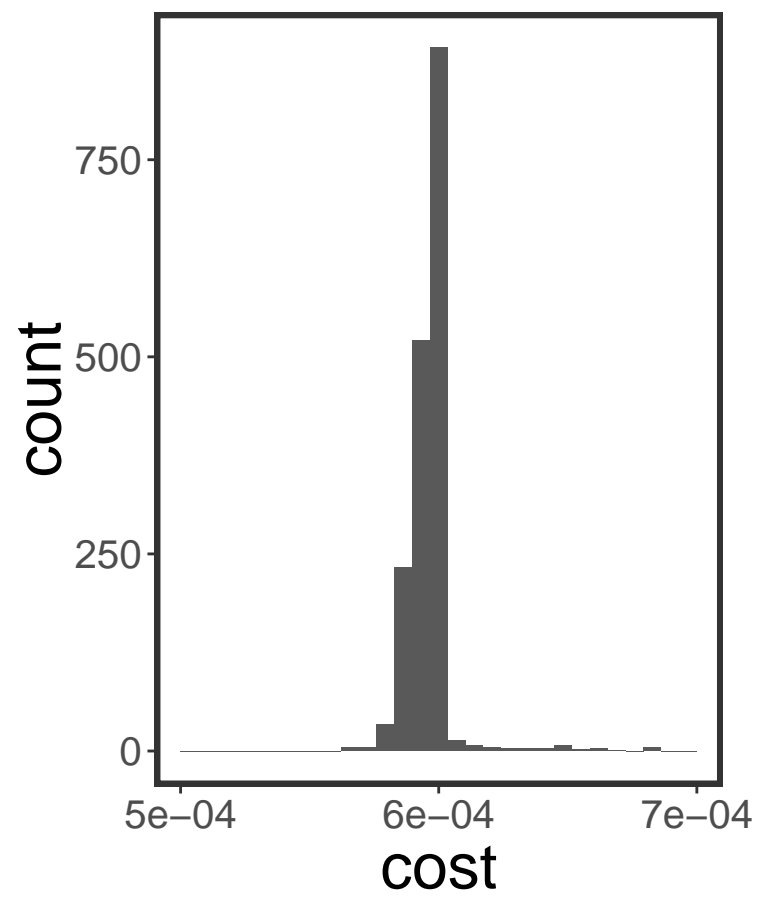

Supplement: evz197_Supplementary_Data [file evz197_supplementary_data.zip › Figure_S7.pdf]

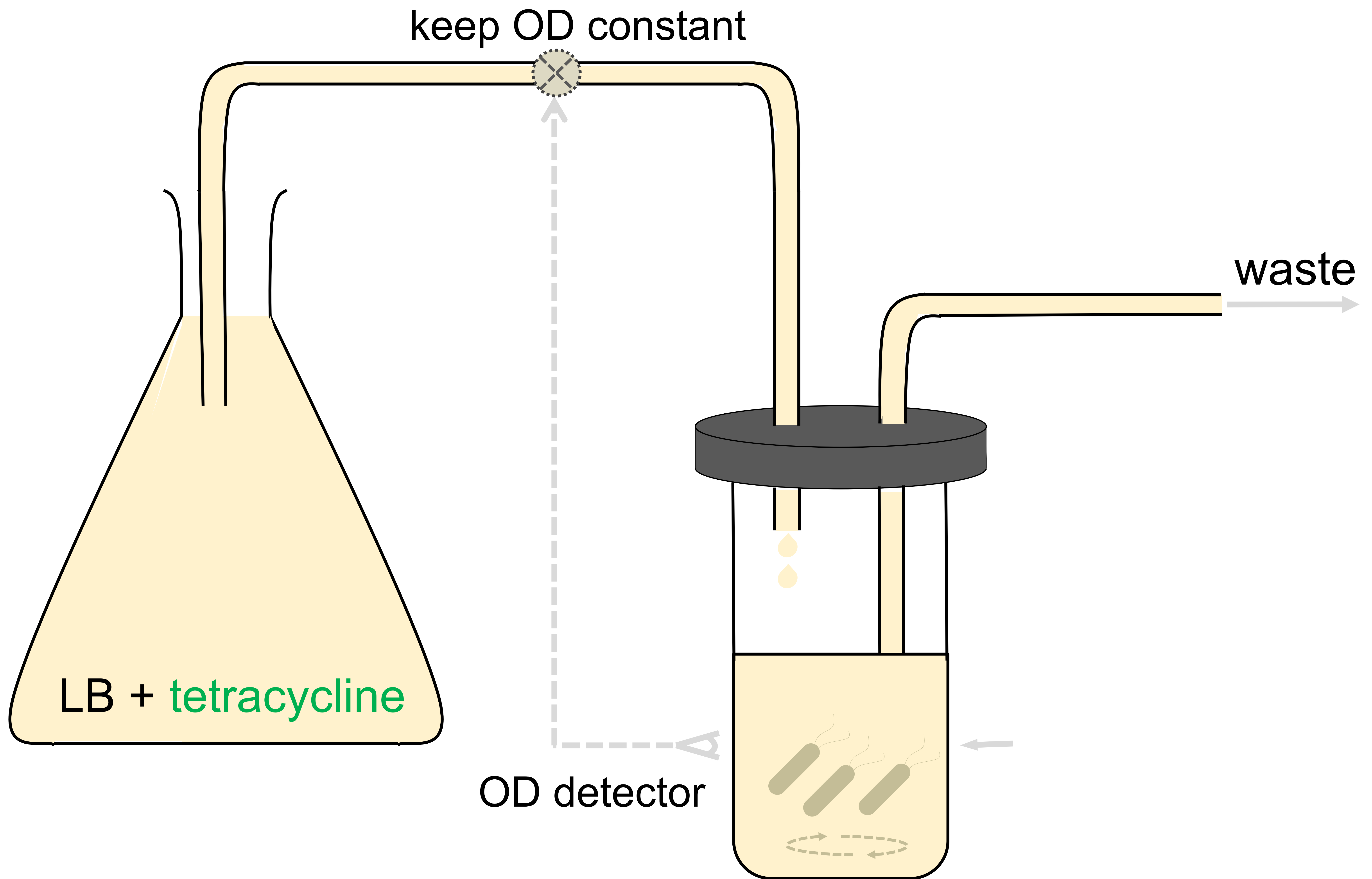

Supplement: evz197_Supplementary_Data [file evz197_supplementary_data.zip › Figure_S8.pdf]
