# Supplementary material for: A High-Resolution View of Adaptive Event Dynamics in a Plasmid
Source: Genome Biol Evol. 2019 Sep 18;11(10):3022–34. doi: 10.1093/gbe/evz197 (PMC6827461; doi:10.1093/gbe/evz197)
Supplement: evz197_Supplementary_Data [file evz197_supplementary_data.zip › Plasmid1 GBE.zip!/main.pdf]

More than one Author with different Affiliations

Author A[1], Author B[1], Author C[1], Author D[2] and Author E[2]

[1]Department of Computer Science, LaTeX University
[2]Department of Mechanical Engineering, LaTeX University

Abstract

$$P(X > S) = 1 - e^{Kmne^{-\lambda S}}$$

$$E = Kmne^{-\lambda S}$$

Hello, here is some text without a meaning. This text should show what a printed text will look like at this place. If you read this text, you will get no information. Really? Is there no information? Is there a difference between this text and some nonsense like "Huardest gefburn"? Kjift – not at all! A blind text like this gives you information about the selected font, how the letters are written and an impression of the look. This text should contain all letters of the alphabet and it should be written in of the original language. There is no need for special content, but the length of words should match the language.

Keywords

Foo, Bar, Baz

Background

3.1 Test

Hello, here is some text without a meaning. This text should show what a printed text will look like at this place. $\sin^2(\alpha) + \cos^2(\beta) = 1$. If you read this text, you will get no information $E = mc^2$. Really? Is there no information? Is there a difference between this text and some nonsense like "Huardest gefburn"? Kjift – not at all! A blind text like this gives you information about the selected font, how the letters are written and an impression of the look. $\sqrt[n]{a} \cdot \sqrt[n]{b} = \sqrt[n]{ab}$. This text should contain all letters of the alphabet and it should be written in of the original language. $\frac{\sqrt[n]{a}}{\sqrt[n]{b}} = \sqrt[n]{\frac{a}{b}}$. There is no need for special content, but the length of words should match the language. $a\sqrt[n]{b} = \sqrt[n]{a^n b}$.

Results

Discussion
