# Supplementary material for: A High-Resolution View of Adaptive Event Dynamics in a Plasmid
Source: Genome Biol Evol. 2019 Sep 18;11(10):3022–34. doi: 10.1093/gbe/evz197 (PMC6827461; doi:10.1093/gbe/evz197)
Supplement: evz197_Supplementary_Data [file evz197_supplementary_data.zip › Plasmid1 GBE.zip!/Figure_1.pdf]

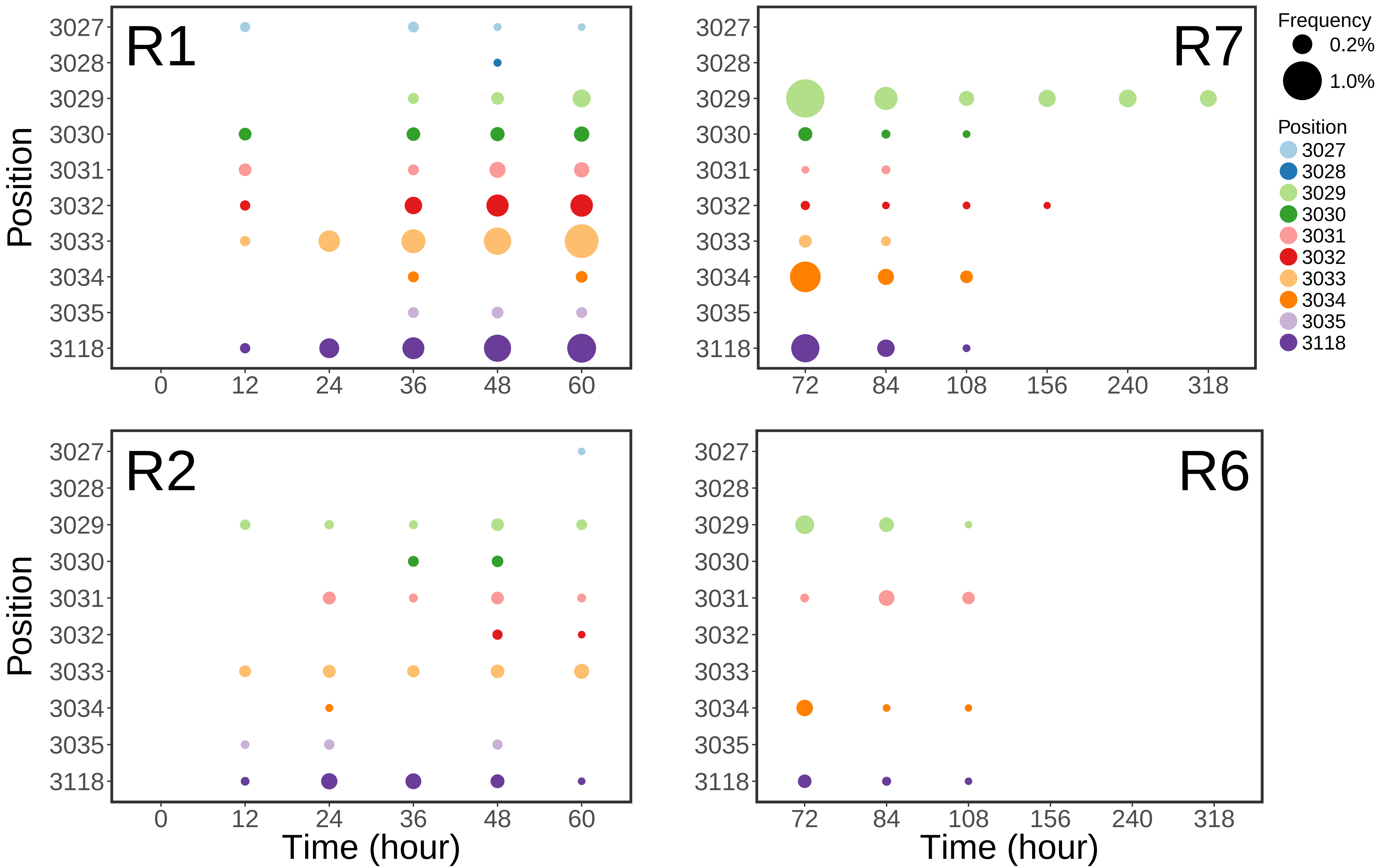
R1
R7
R2
R6
Position
Time (hour)
3027
3028
3029
3030
3031
3032
3033
3034
3035
3118
0
12
24
36
48
60
72
84
108
156
240
318
Frequency
0.2%
1.0%
Position
