# Supplementary material for: A High-Resolution View of Adaptive Event Dynamics in a Plasmid
Source: Genome Biol Evol. 2019 Sep 18;11(10):3022–34. doi: 10.1093/gbe/evz197 (PMC6827461; doi:10.1093/gbe/evz197)
Supplement: evz197_Supplementary_Data [file evz197_supplementary_data.zip › Plasmid1 GBE.zip!/Figure_2.pdf]

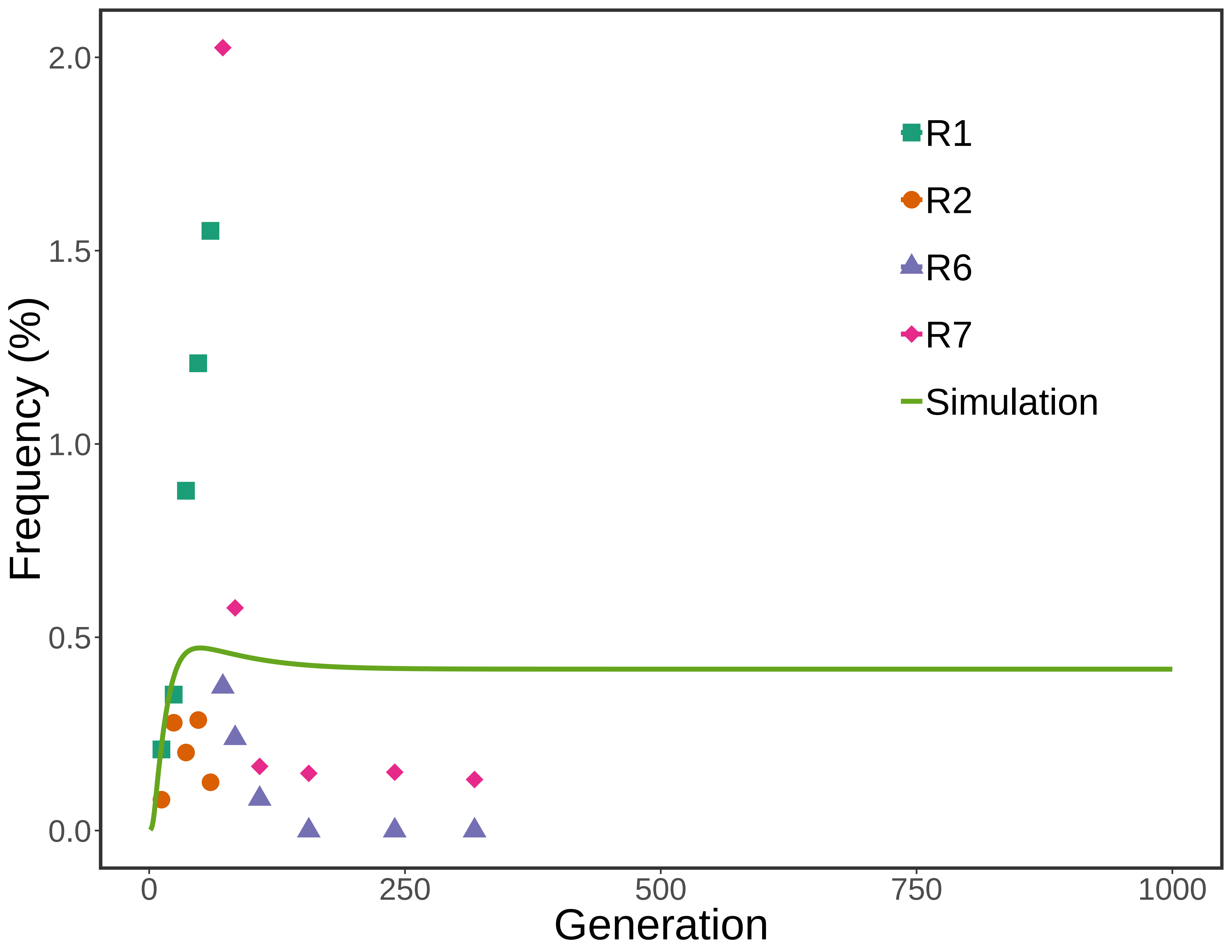

2.0
1.5
1.0
0.5
0.0
Frequency (%)
0
250
500
750
1000
Generation
R1
R2
R6
R7
Simulation
